# Taxonomy, Sequence Variance and Functional Profiling of the Microbial Community of Long-Ripened Cheddar Cheese Using Shotgun Metagenomics

**DOI:** 10.3390/microorganisms11082052

**Published:** 2023-08-10

**Authors:** Hassan Mahmoud Mohamed, Zoha Barzideh, Myra Siddiqi, Gisèle LaPointe

**Affiliations:** 1 Dairy at Guelph, Department of Food Science, University of Guelph, Guelph, ON N1G 2W1, Canada; 2 Faculty of Computer and Artificial Intelligence, Benha University, Banha 13518, Egypt

**Keywords:** Cheddar cheese, metagenomics, viable bacteria, microbiota, microbial interactions

## Abstract

Shotgun metagenomic sequencing was used to investigate the diversity of the microbial community of Cheddar cheese ripened over 32 months. The changes in taxa abundance were compared from assembly-based, non-assembly-based, and mOTUs2 sequencing pipelines to delineate the community profile for each age group. Metagenomic assembled genomes (MAGs) passing the quality threshold were obtained for 11 species from 58 samples. Although *Lactococcus cremoris* and *Lacticaseibacillus paracasei* were dominant across the shotgun samples, other species were identified using MG-RAST. NMDS analysis of the beta diversity of the microbial community revealed the similarity of the cheeses in older age groups (7 months to 32 months). As expected, the abundance of *Lactococcus cremoris* consistently decreased over ripening, while the proportion of permeable cells increased. Over the ripening period, the relative abundance of viable *Lacticaseibacillus paracasei* progressively increased, but at a variable rate among trials. Reads attributed to *Siphoviridae* and *Ascomycota* remained below 1% relative abundance. The functional profiles of PMA-treated cheeses differed from those of non-PMA-treated cheeses. Starter rotation was reflected in the single nucleotide variant profiles of *Lactococcus cremoris* (SNVs of this species using mOTUs2), while the incoming milk was the leading factor in discriminating *Lacticaseibacillus paracasei*/*casei* SNV profiles. The relative abundance estimates from Kraken2, non-assembly-based (MG-RAST) and marker gene clusters (mOTUs2) were consistent across age groups for the two dominant taxa. Metagenomics enabled sequence variant analysis below the bacterial species level and functional profiling that may affect the metabolic interactions between subpopulations in cheese during ripening, which could help explain the overall flavour development of cheese. Future work will integrate microbial variants with volatile profiles to associate the development of compounds related to cheese flavour at each ripening stage.

## 1. Introduction

Cheddar cheese production in Canada totaled 164 million kilograms in 2022 [1], making up about 20% of all the cheese produced, while in 2016, Canadians consumed an average of 15.07 kg of cheese [2]. Ripening of hard cheeses represents a significant investment in equipment and efforts in controlling temperature and relative humidity to ensure consistent organoleptic properties over extended periods [3], sometimes exceeding 3 years. Predicting ripening outcomes would thus facilitate decision-making by cheesemakers, reducing losses and waste [4].

The microbiota of ripening Cheddar cheese is dominated by lactic acid bacteria, which are either added as starter cultures (SLAB) and adjunct cultures or originate from the production and processing environments (nonstarter or NSLAB). The SLAB used for Cheddar cheese production contain mesophilic lactic acid bacteria, which optimally grow between 28 °C and 32 °C. Cheddar starter cultures consist of one or more strains, mainly of *Lactococcus cremoris* and *Lactococcus lactis* subsp. *lactis*. Starter cultures may also contain bacteria that can utilize the citrate in milk (*L. lactis* subsp. *lactis* biovar *diacetylactis* or *Leuconostocaceae*). The primary role of starter cultures in Cheddar cheese production is the production of lactic acid [3]. After curd formation and pressing, starter bacteria reach high numbers, but their viability decreases over ripening due to lactose depletion, salt addition, low pH and temperature. The rate of this decrease depends on such factors as the lysis of the starter or the use of adjunct ingredients [5].

The NSLAB are adventitious bacteria from dairy production and processing environments that contribute significantly to flavour production during ripening. NSLAB may be present in curd at low concentrations (2 to 3 log cfu/g), but throughout the first few months of ripening, their populations can grow by 4 to 6 orders of magnitude [6]. Depending on the rate of decrease in starter bacteria viability, NSLAB may predominate the viable microbiota of cheese for most of the ripening process of hard and semi-hard cheeses, such as Cheddar, Emmental, and Gruyere [7]. In particular, *Lacticaseibacillus* species replicate and release enzymes that contribute to the ripening of hard cheeses (reviewed in [8]). The strain-specific metabolic capacity of *Lacticaseibacillus* species has been demonstrated through physiological and genetic approaches [7,8], resulting in modifications of the volatile profile of the ripened cheese, particularly in terms of fruity/green/nutty notes (3-methylbutanal and 3-methyl-1-butanol) or fruity/flower aromatic notes (1-hexanol). Thus, relating this phenotypic variation to genotype will help delineate the ripening outcome.

Propidium monoazide (PMA) is a DNA-intercalating dye that can bind free DNA or diffuse into cells with compromised membranes. Once inside the cells, PMA can be covalently cross-linked by exposure to ultraviolet light, which then strongly inhibits PCR amplification of DNA from these permeable cells [9]. Therefore, treatment of the cells with PMA before DNA extraction restricts the PCR reactions to DNA from the presumably viable bacteria with intact cell membranes. A reduction in PMA-treated DNA compared to total DNA would mean fewer intact viable cells and thus more permeable cells. When the number of permeable cells increases, PMA can enter and prevent amplification, thus leading to a decrease in amplified DNA after PMA treatment compared to total DNA. Even though membrane integrity is not proof of cell viability, selecting intact cells would provide a view of the presumably active bacterial community. This method has been validated for several bacterial species, including *Lactococcus lactis* and *Lactobacillus* [10,11], in pure culture and in a cheese matrix [12].

The composition of the microbial community can vary from batch to batch of cheese due in part to the milk microbiota, equipment cleanliness, time and location (surface versus core). These changes may influence the consistency of the organoleptic properties of the final product due to the enzymatic activity of the microbes [13,14]. For microbial community profiling, a common metagenetic approach is 16S rRNA gene sequencing (amplicon sequencing), which targets the classification of PCR amplicon sequences of a specific region of the small subunit ribosomal RNA gene. Our previous study applied 16S rRNA gene amplicon sequencing to ripened cheese samples for up to 32 months. The viable population of *Lactococcus lactis* gave way to *Lacticaseibacillus* spp. after 7 months, while potential defect-causing bacterial genera became more frequent as the cheese aged [15]. 16S rRNA gene sequencing provided an understanding of genus-level population dynamics in ripened cheese samples. However, this method has limitations concerning the resolution of species and intra-species sequence variation.

The availability of gene and genome sequences has enabled the targeted detection of specific cheese microbes over the ripening period [4]. Integrated systems biology is needed to combine the multiple perspectives of post-genomics technologies to elucidate the metabolic interactions among microorganisms [6]. Shotgun metagenomics represents a deeper resolution approach than 16S rRNA gene profiling for characterizing microbial communities within cheese [16]. This technology, coupled with recent advances in bioinformatics, enables the determination of genotypic variation within species [17], which helps to delineate metabolic potential and diversity. This leads to inferring functional information [18], enriching taxonomic profiling with unprecedented depth up to species- and intra-species-level variation of microorganisms that could affect the flavour of cheese, and discovering phage abundance [19]. However, a significant challenge facing this approach is the difficulty in assembling genomes from highly diverse sequences of cheese microbiota using as a reference the available genomes deposited in widely used public databases [20].

This study aims to identify the diversity and successional dynamics of cheese microbial communities in long-ripened Cheddar cheese produced in a conventional industrial cheese-making processing facility in Canada over a three-year period using shotgun metagenomics. Both assembly-based and non-assembly methods were used to analyze the metagenomic sequencing data to provide a comprehensive view of genomic potential, interaction dynamics, and the ability to predict microbial functions enriched during cheese ripening. A main contribution of this study is comparing a non-assembly-based approach using MG-RAST, an assembly-based strategy using BV-BRC (3.28.21), and a mOTUs2 hybrid approach using marker genes for analyzing metagenomic sequences. The results will be applicable to further defining the persistence of specific sequences of the major taxa at later ripening stages and their contribution to product quality in terms of organoleptic and sensory properties.

## 2. Materials and Methods

### 2.1. Cheese Production and Sampling

Cheddar cheeses were produced in an industrial facility using conventional cheese-making methods; curd was cooked at 38.5 °C, pitched at pH 6.15, milled at pH 5.35, and salted at 2.7% (*wt*/*wt*). The same milk was used in 6 vats per trial using two different starters per trial (3 vats per starter) with a rotation of 7 undefined starter mixes containing mostly *Lactococcus cremoris* (at least 90%) with under 10% *L. lactis*. Each starter was repeated on average 1 or 2 times over the 5 trials. Starter cultures were inoculated at 0.5% *v*/*wt* (volume of 115 ± 12.3 L per 23,000 kg of milk). A total of 58 samples (51 non-PMA-treated DNA and 7 PMA-treated DNA) from separate vats were sampled from the 5 cheese trials at six time points over 32 months (m) of ripening (Table 1). All cheeses were aged in the same facility, and cheese samples were taken from the ripening room at 0–1 m, 3–6 m, 7–10 m, 18–20 m, 24 m and 30–32 m time points. Samples at day zero were in the form of cheese curd; at 1 month, they were cheese plugs; and samples at three months and older were blocks of 20-pound cuts that were obtained from the 640-pound mother block. The 20-pound block was cut into half so that every 10-pound block had two or three exterior and interior faces. Sections of the 10-pound block, including internal and external faces, were ground before homogenization in a buffer as described below.

### 2.2. Cheese Homogenate Preparation for Bacterial Enumeration and DNA Extraction

Ten grams of each cheese sample were suspended in 90 mL of 45 °C sterile 2% (*wt*/*vol*) trisodium citrate solution and homogenized in stomacher bags (Fisher Scientific, Mississauga, ON, Canada) using a Seward Stomacher^®^ 400 Circulator for 5 min at 260 rpm. Ten mL of the cheese homogenate was centrifuged at 10,000× *g* for 10 min at room temperature. After centrifugation, the supernatant was removed, and the fat layer was cleared as much as possible with a sterile cotton swab. Once the fat layer was cleared, cells were suspended in 1 mL of a 2% sodium citrate solution for washing. The cell suspension was centrifuged at 10,000× *g* for 5 min and the supernatant was removed. This step was repeated 3–4 times until no fat was left in the tube. The resulting cell pellet was used for DNA extraction.

Duplicate pellets were used for DNA extraction both with and without PMA treatment (for trials 1 and 2 only for ages 7 m and older). PMA treatment of cell pellets was performed following the protocol described by Desfossés-Foucault et al. [12], with some modifications. Each pellet was suspended in 500 μL of 0.1% buffered peptone water and treated with 5 μL of 2.5 mM PMA solution (PMAxx dye, Biotium, Fermont, CA, USA) diluted in nuclease-free water. Samples were incubated on ice in the dark for 15 min. Samples were then exposed to PMA UV light (PhAST Blue, Genius) for 15 min and placed on ice in the dark for another 15 min. The PMA-treated cell suspensions were centrifuged at 10,000× *g* for 5 min, followed by washing the cell pellets with 500 μL of a 2% *w*/*v* sodium citrate solution, followed by centrifugation at 10,000× *g* for 1 min, and discarding the supernatant.

DNA extraction was carried out on cell pellets with and without PMA treatment using the Invitrogen PureLink Microbial DNA Purification kit following the manufacturer’s instructions (Invitrogen Canada Inc., Burlington, ON, Canada) with some modifications. Bacterial pellets were suspended in 800 μL of lysis buffer and transferred to a 2-milliliter microtube containing 0.3 g of zirconium beads (1-mm diameter). One hundred μL of lysis enhancer was added, vortexed briefly and incubated at 75 °C for 10 min. Tubes were shaken for 10 min at maximum speed on the vortex mixer and then centrifuged at 14,000× *g* for 2 min.

Up to 500 μL of the supernatant was transferred to a clean microcentrifuge tube, avoiding the bead pellet and any debris. Nine hundred μL of binding buffer were added, vortexed briefly, loaded onto a spin column tube assembly, and centrifuged at 14,000× *g* for 1 min. The column was washed using 500 μL of wash buffer, and DNA was eluted using 50 μL of elution buffer, followed by storage at −20 °C until further analysis. For cheese samples with a lower cell count, mostly samples older than one year, three to six cell pellets were treated separately up to the step where the DNA was loaded on the DNA binding column. The extracted nucleic acids from all cell pellets were loaded on a single column, washed, and collected as one sample.

Over the 5 trials, we obtained sufficient DNA from 51 cheese samples that were not treated with PMA (Table 1). Sufficient DNA was obtained from a total of seven cheese samples that were treated with PMA (3 at Age 3, 3 at Age 6 and 1 at Age 7) from trials 1 and 2 only.

### 2.3. Shotgun Metagenomics Sequencing

A minimum of 10 μL of DNA with a concentration between 20 and 300 ng/μL, representing at least 200 ng of DNA per sample, was used for shotgun metagenomics sequencing of cheese samples from selected ripening time points. Libraries were prepared using the NEB Ultra II DNA kit following the procedures described in the NEBNext^®^Ultra^TM^ II DNA Library Prep Kit for Illumina^®^ (E7645, E7103) (Illumina 2020, Illumina, San Diego, CA, USA). All samples were sequenced on Illumina HiSeq 2500 (Illumina, San Diego, CA, USA) high throughout an 8-lane flow cell for paired ends of 2 × 125 bp. Using this system together with the Rapid Run mode flow cell, a total of 150–170 million reads were generated, facilitating multiplexing samples in the same lane (reducing the total cost of the experiment per sample).

### 2.4. Data Analysis

The bioinformatics data analysis was carried out using the R programming language with three approaches: assembly of reads, non-assembled reads, and a hybrid shotgun metagenomics approach with a set of marker genes.

#### 2.4.1. Assembly-Based Metagenomic Sequence Analysis

The taxonomic classification for each cheese sample was computed using BV-BRC 3.28.21. Raw reads from metagenomic samples were assigned to taxonomic bins using Kraken2. This method takes metagenomic DNA sequences using an alignment of k-mers and assigns them to taxonomic labels.

Metagenomic assembled genomes (MAGs) were constructed using a metagenomic binning pipeline (BV-BRC 3.28.21) [21]. Raw reads were assembled using MetaSpades at a minimum contig length of 300 bp with a minimum coverage of 5%. Bacteria/Archaea and Viruses were chosen as organisms of interest. The RASTtk [22] pipeline was used for annotation. Two internal BV-BRC quality tools were used: EvalG7 [21], which computes the completeness and contamination of MAG annotation, and EvalCon7 [21] to evaluate the consistency of MAG annotation. The binning quality threshold was established at 80% completeness, 10% contamination, and 87% consistency. CheckV [21] was used to estimate the completeness of non-bacterial contigs (i.e., viruses).

The metagenomic binning was carried out with the following BV-BRC pipeline: First, all occurrences of *pheS* in the sample were identified by comparing the contigs in the sample against a small database of *pheS* sequences using BLAST. Next, the supervised binning method sorted contigs into draft genomes by comparing them to the reference set of genomes to place them into bins. To assign reference genomes, the binning service compares the DNA sequences of the *pheS* instances in each bin to the reference set and chooses the closest match. Two bins are merged if their reference genomes belong to the same species. Contigs were then placed into the bin belonging to a reference genome set if there were at least 10 discriminating protein 12-mers in common with that reference genome and no other reference genome had more discriminating protein 12-mers in common with the contig being binned. This approach identified a reference genome and, instead of analyzing the whole genomes, quantified the read coverage of genes that were found to be clade-specific based on analyzing current reference genome databases. If such marker genes occur only once per genome, then the resulting read coverages do not need to be normalized by copy number or genome length. However, a downside to any method depending on prior knowledge of genome sequences is that uncharacterized taxa remain unaccounted for, which can lead to inaccurate relative abundance estimates at species-level resolution. Missed taxa include organisms referred to as ‘unknown’ species that may be detected but remain difficult to quantify using standard methods and up-to-date genome databases.

#### 2.4.2. Non-Assembly-Based Metagenomic Sequence Analysis

Annotation of unassembled DNA sequences was conducted with Metagenomics Rapid Annotation (MG-RAST data analysis pipeline available at https://www.mg-rast.org (accessed on 20 November 2021)). This approach relies on classifying sequencing reads using publicly available and taxonomically annotated reference genome sequences of ‘known’ species. The resulting read abundance distributions require subsequent normalization by genome length to deduce the relative abundances of each species. We overcome the genome length normalization step because, in our samples, *Lactococcus* spp. and *Lacticaseibacillus* spp. have similar genome lengths of 2.51 Mb and 2.95 Mb, respectively, and constitute the major proportion compared to the other species. Normalization was instead carried out per sample.

The quality control step included the removal of low-quality reads, filtering of contaminants, and trimming of adapter sequences. The default parameters used for quality control were minimum sequence length = 50 bp, maximum ambiguous base pairs = 5, maximum homopolymer length = 6 bp, maximum error rate = 1%, maximum percent of mismatches allowed in overlap region = 10%, and contamination screening using NCBI BLAST against the NCBI RefSeq database (v. 72).

Taxonomic classification with MG-RAST used the M5NR (non-redundant) database, which combines data from several databases, including GenBank, UniProt, and RefSeq. The parameters applied for taxonomic classification were as follows: minimum alignment length = 15 bp, minimum percent identity = 60%, minimum bit score = 50, maximum e-value = 1 × 10^−5^, and best-hit classification using the LCA (lowest common ancestor) algorithm.

Functional annotation with MG-RAST used the SEED database, which is a hierarchical system that classifies genes into subsystems based on their functions. The default parameters applied for functional annotation were minimum alignment length = 30 bp, minimum percent identity = 60%, minimum bit score = 50, maximum e-value = 1 × 10^−5^, best-hit classification based on closest subsystem, and subsystem level 3.

#### 2.4.3. Hybrid Sequence Analysis Based on 10 Marker Genes

The marker gene-based operational taxonomic units tool (mOTUs2, available at https://github.com/motu-tool/mOTUs (accessed on 15 April 2022)) was used for taxonomic profiling, which conducts functional profiling on the basis of universally occurring, protein-coding, single-copy phylogenetic marker genes (MG) to capture and quantify microbial taxa at species-level resolution in metagenomic samples. mOTUs2 consolidates data from >3100 metagenomic samples and builds on ten universally occurring, protein-coding, single-copy phylogenetic marker genes (MGs) from both known and unknown species, the latter of which are extracted from existing metagenomes, enabling higher taxonomic resolution and more accurate quantification of species when profiling new microbial communities. SNV analysis based on marker gene COGs [23] for microbial population analyses was also conducted using mOTUs2. The computation of metagenomic SNV profiles to study microbial population differences below the species level is both resource-intensive and time-consuming when using methods based on whole reference genome sequences. The use of mOTUs2 provides a fast and efficient alternative for profiling the abundance of species and SNVs in microbial communities. In addition to the improved efficiency, mOTUs2 enables studying differences in strain populations of species that currently lack a representative genome sequence. This may be particularly relevant for disease-associated species and biomes, for which only a few reference genomes are available.

The raw sequencing reads were quality-filtered and preprocessed to remove adapters, low-quality reads, and contaminating sequences using the Trimmomatic software. The preprocessed reads were mapped to a reference database of bacterial genomes using the Bowtie2 software. The mapped reads were processed to call SNVs at the nucleotide level using the VarScan2 software. The default parameters for SNV calling in mOTUs2 were a minimum read depth of 10 reads, a minimum variant frequency of 90% in the reads, and a minimum base quality score of 20. The SNVs were used to identify and quantify bacterial taxa in the metagenomic sample using the Motus2 algorithm, which combines the SNV information with the read mapping information. For SNV analysis with these parameters, two samples (S8 and S10) had insufficient coverage for *Lactococcus cremoris* and three samples for *Lacticaseibacillus paracasei*/*casei* (S6, S32 and S42), so they were filtered out for the PCoA analysis. Heatmaps were generated using hierarchical clustering in *R* of the SNV profiles of samples based on the Ward criterion and Manhattan distance.

## 3. Results

The microbial communities of fifty-eight ripened Cheddar cheese samples spanned across six age groups and five trials, of which seven samples were PMA-treated (from two trials). Two cheese samples from the same trial were ripened until 30–32 m, while one of them was PMA treated; eight samples from two trials were ripened for 24 m, where three samples were PMA treated; three samples from the same trial were ripened until 18–20 m; eighteen samples from three trials were ripened to 7–10 m, among which three samples were PMA treated; twenty-one cheese samples from four trials were 3–6 m old; and six samples of 0–1 m were from the same trial. Shotgun sequencing was not performed on cheeses in Age group 4 (12–16 m).

The number of reads per sample, quality screening, and sequencing depth are shown in Supplementary Tables S1 and S2. Across age groups, more than 93.03% of sequences passed quality control (QC) and were assigned to protein sequences. On average, less than 7% of the sequences passing QC were unknown across seven PMA-treated samples and fifty-one non-PMA-treated samples. Sequence analysis methods used assembly-based, non-assembly-based, and mOTUs2-hybrid approaches.

### 3.1. Assembly-Based Sequence Analysis

A total of 110 MAGs out of 165 showed quality criteria of completeness ≥ 80%, fine consistency ≥ 87%, contamination ≤ 10%, and a single PheS protein of reasonable size (Supplementary Figure S2a,b), and the number of good-quality MAGs was six, twenty-one, forty-two, six, thirty-one and four for Ages 1, 2, 3, 5, 6 and 7, respectively. The taxonomic profiling of fifty-one cheese samples without PMA treatment resulted in MAGs from eleven species belonging to nine genera at ≥0.1% relative abundance in ≥10% of samples (the defined threshold of prevalence). Overall, the mean abundance of Bacteria, Eukaryota and viruses was 98.90%, 0.09% and 0.76% in non-PMA samples, and 99.45%, 0.11%, and 0.39%, respectively, in PMA samples.

The 110 MAGs with good binning quality were classified into two phyla: Firmicutes and Proteobacteria. The MAGs with good quality binning were *Lactococcus cremoris*/*lactis*, *Lacticaseibacillus*, *Weissella*, *Streptococcus*, *Secundilactobacillus*, *Latilactobacillus*, *Paucilactobacillus*, *Pediococcus*, and *Enterobacter* (Supplementary Figure S2b). Although the genus frequency changed over time, MAGs for all nine genera were assembled from 24-month cheeses (Age 6). The ranking of age groups based on the total number of MAGs detected was Age 3, Age 6, Age 2, Age 5, Age 7 and Age 1 (Supplementary Figure S2a). Six genera were found in the seven PMA-treated DNA samples (*Lactococcus*, *Lacticaseibacillus*, *Secundilactobacillus*, *Streptococcus*, *Weissella*, *Pediococcus*, and *Enterobacter* entailing 10 species).

**Figure 1:**
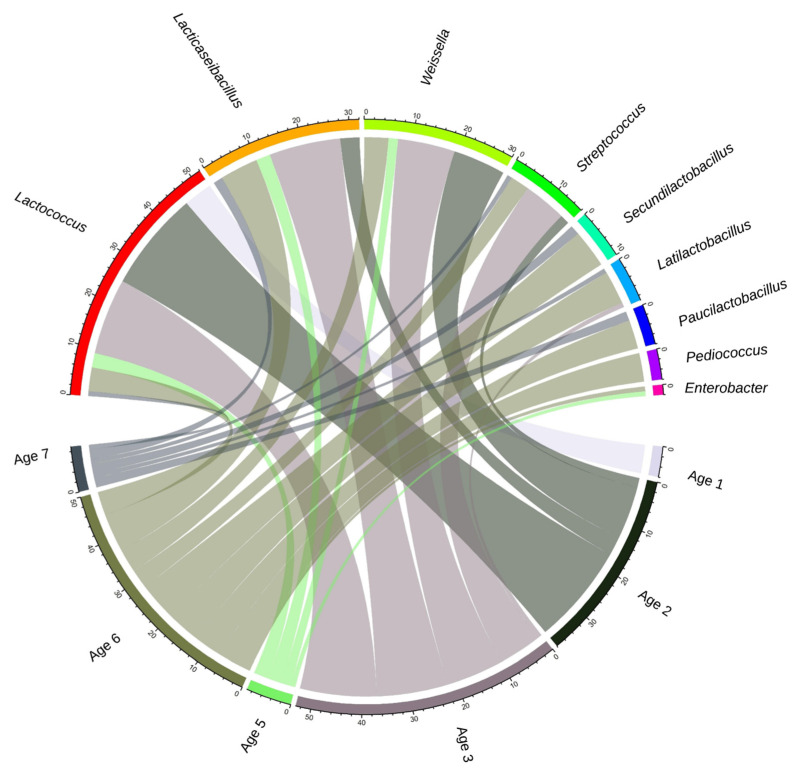
Radial visualization of the number of MAGs with good binning quality in 9 genera and the age group of the source cheese. The size of each chord shows the weight of each dominant species in the corresponding age group.

At the beginning of ripening (Age 1; 0–1 m), 100% of good-quality binned MAGs were *Lactococcus* (Supplementary Figure S2). MAGs of *Lactococcus* decreased in later months as ripening advanced, representing 28% of MAGs overall. Out of a total of 52 *Lactococcus cremoris*/*lactis* MAGs, 31 passed the quality check and 74% of these contained prophage sequences. *Lacticaseibacillus* was the most abundant genus, starting from 3 to 6 m, with 22% of MAGs overall, while *Weissella* was the second most abundant NSLAB genus, representing 20% of MAGs. As ripening progressed, more MAGs were binned to *Lacticaseibacillus* and *Weissella* (Supplementary Figure S2). Out of twenty-four high-quality *Lacticaseibacillus* spp. MAGS, seventeen belonged to *L. paracasei,* while seven were binned with *L. casei*. There were no MAGs binned with *L. rhamnosus*. *Streptococcus* MAGS were obtained at Ages 2, 3, and 6, representing 11% of good-quality MAGs. The association linking species and ages reveals that the MAGs obtained were higher in species diversity from cheeses at Ages 3 and 6 compared to Ages 1, 2 and 5 (Figure 1).

### 3.2. Non-Assembly-Based Sequence Analysis Using MG-RAST

**Figure 2:**
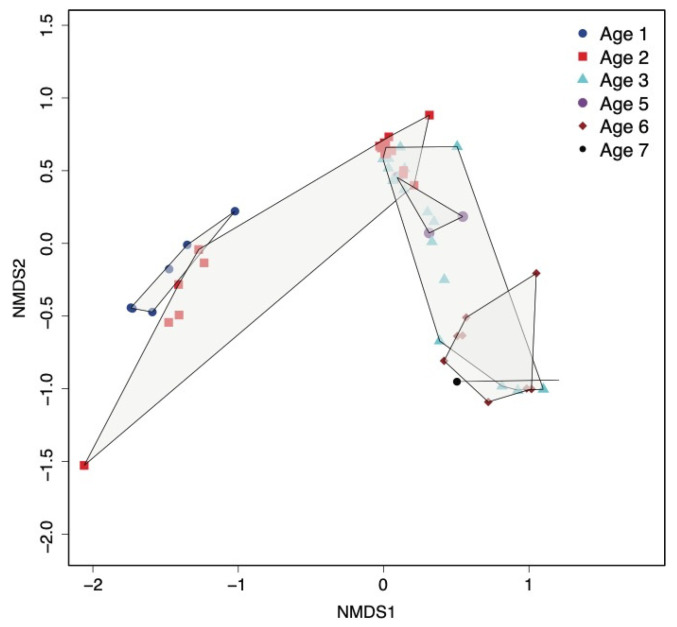
Multivariate nonlinear multidimensional scaling (NMDS) analysis of the Bray–Curtis dissimilarity (beta diversity) between samples, calculated using the relative abundance of OTUs (non-assembly-based analysis using MG-RAST). Samples of each age group are bounded by a polygon shape delineating sample clustering by age.

The non-assembly-based data analysis by MG-RAST was used to cluster samples based on the underlying abundance of OTUs profiled at each age group (Figure 2). Cheeses of Age 2 are widely spread, so some samples are closer to Age 1 cheeses while the majority overlap with Age 3 samples. This separation is mainly due to the relative abundance of *Lactococcus* spp., which is higher in cheeses at Age 1 but only higher in six cheeses at Age 2 (those made with starter R6). The spread of samples of Age 3 is mainly due to the relative abundance of *Lacticaseibacillus* spp., with a group of 10 samples with low abundance (under 20%) separated from five samples with a mid-to-high abundance of *Lacticaseibacillus* spp. (from 31% to 71%), which mostly overlap with Age 6 cheeses containing a similarly high abundance of *Lacticaseibacillus* spp. (Figure 2). The non-metric fitting between ordination distance and observed dissimilarity resulted in *R*^2^ = 0.996 (Supplementary Figure S3).

**Figure 3:**
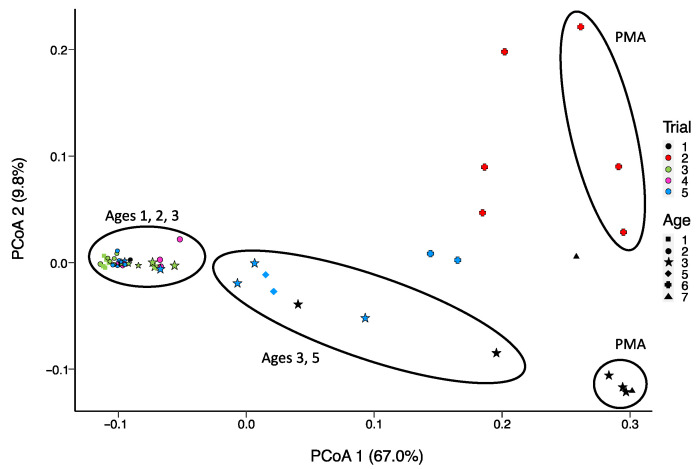
Principle Coordinate Analysis (PCoA) of the Bray–Curtis dissimilarity between samples in terms of frequencies of predicted function categories (subsystem level; MG-RAST) based on non-assembled reads from shotgun sequencing of DNA extracted from aged Cheddar cheese, untreated or treated with PMA (7 samples grouped with ellipses and identified with PMA).

**Figure 4:**
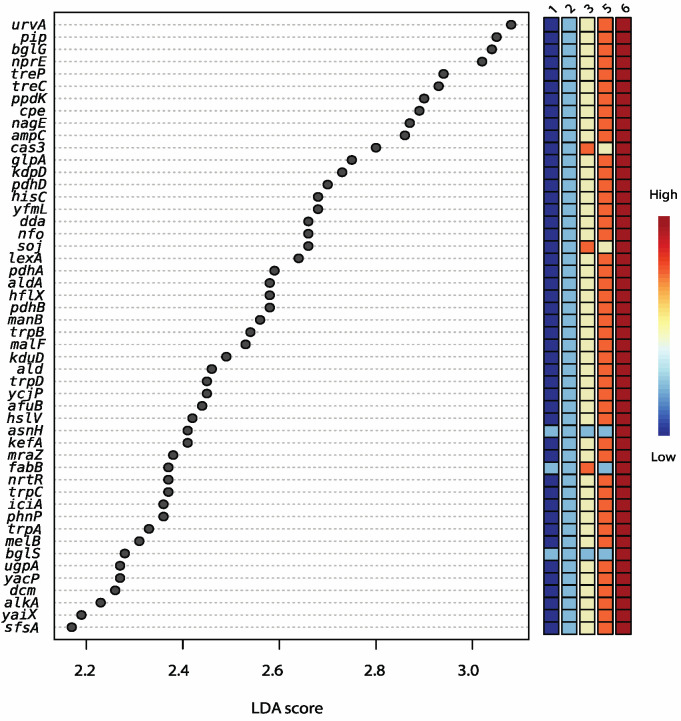
LDA scores from LefSe analysis showing the significantly enriched (*p* ≤ 0.05) predicted subsystem functions based on non-assembled reads from shotgun sequencing of DNA extracted from aged Cheddar cheese that were not treated with PMA (samples from 5 out of 7 age groups showed significant distinct features; columns show the colour gradient from Ages 1 to 6).

PMA-treated samples can be distinguished from non-PMA-treated samples regardless of trial and age group based on predicted functional categories at the subsystem level (Figure 3). Age 3 samples are widely distributed across the *x*-axis, grouping with Age 5 cheeses. Functions that were significantly enriched with age include genes involved in DNA repair and potential acid resistance (*uvrA*), β-glucoside starvation (*bglG*), osmoprotection (trehalose operon), peptide metabolism (*pip*), amino acid conversion (*hisA*, *trpAC*), alternative carbon source metabolism (aminosugar transport and degradation, for example), as well as other stress conditions (*hflX*) (Figure 4).

### 3.3. Comparison of Non-Assembly-Based Sequence Analysis with Marker Gene Analysis

**Figure 5:**
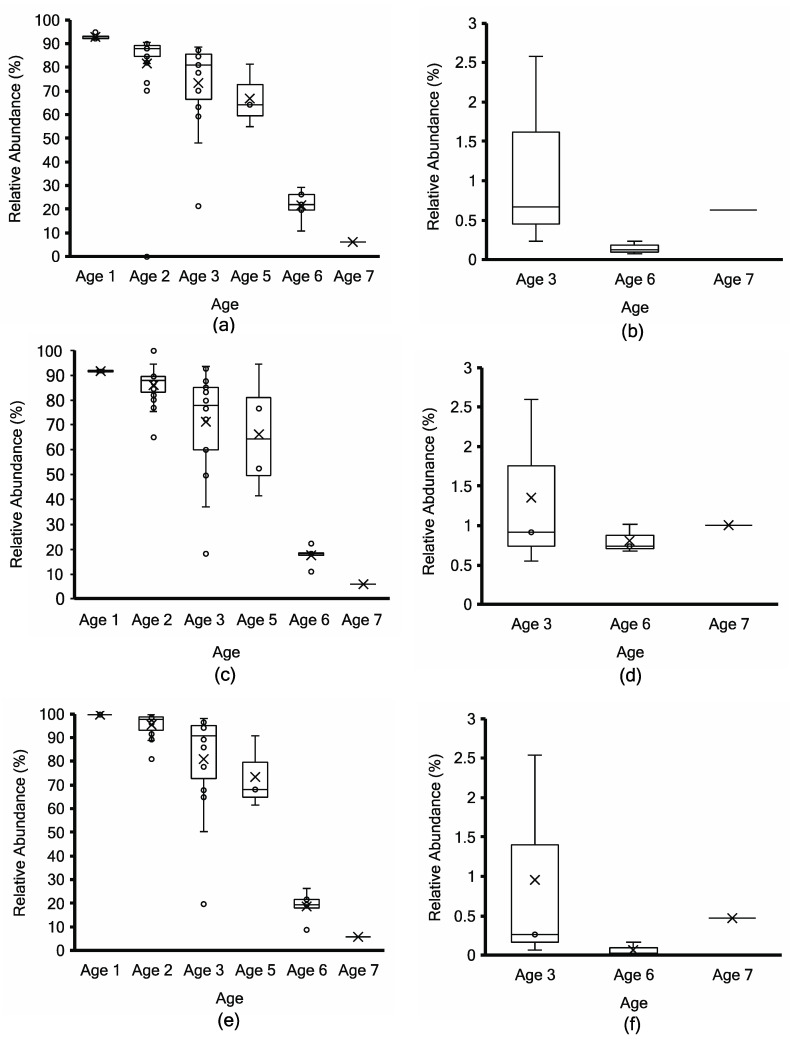
*Lactococcus cremoris*/*lactis* taxonomy profiling by Kraken2 (top; (**a**,**b**)) vs. non-assembly-based taxonomy profiling by MG-RAST (middle; (**c**,**d**)) vs. mOTUs2 marker gene cluster-based taxonomy profiling (bottom; (**e**,**f**)) for non-PMA-treated (**a**,**c**,**e**) vs. PMA-treated (**b**,**d**,**f**) aged Cheddar cheese samples. Each box consists of the 75% quartile with a horizontal line representing the median value, accompanied by whiskers showing the 95% confidence interval. The mean value of relative abundance is denoted by “×”, while individual samples are denoted by “o”.

**Figure 6:**
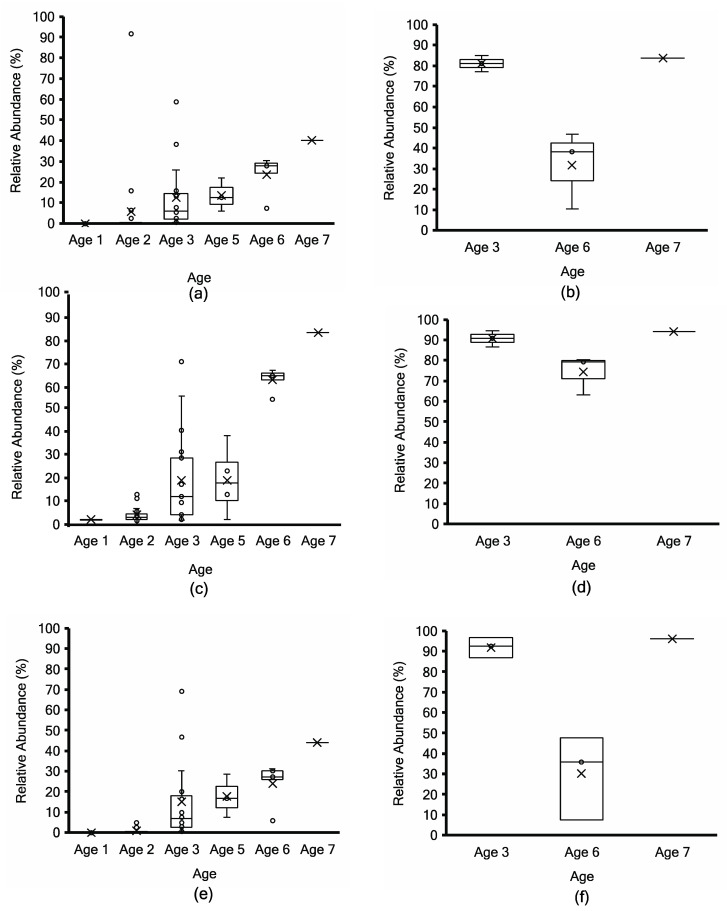
*L. paracasei*/*casei* taxonomy profiling by Kraken2 (top; (**a**,**b**)) vs. non-assembly-based taxonomy profiling by MG-RAST (middle; (**c**,**d**)) vs. mOTUs2 marker gene cluster-based taxonomy profiling (bottom; (**e**,**f**)) for non-PMA-treated (**a**,**c**,**e**) vs. PMA-treated (**b**,**d**,**f**) aged Cheddar cheese samples. Each box consists of the 75% quartile with a horizontal line representing the median value, accompanied by whiskers showing the 95% confidence interval. The mean value of relative abundance is denoted by “×”, while individual samples are denoted by “o”.

**Figure 7:**
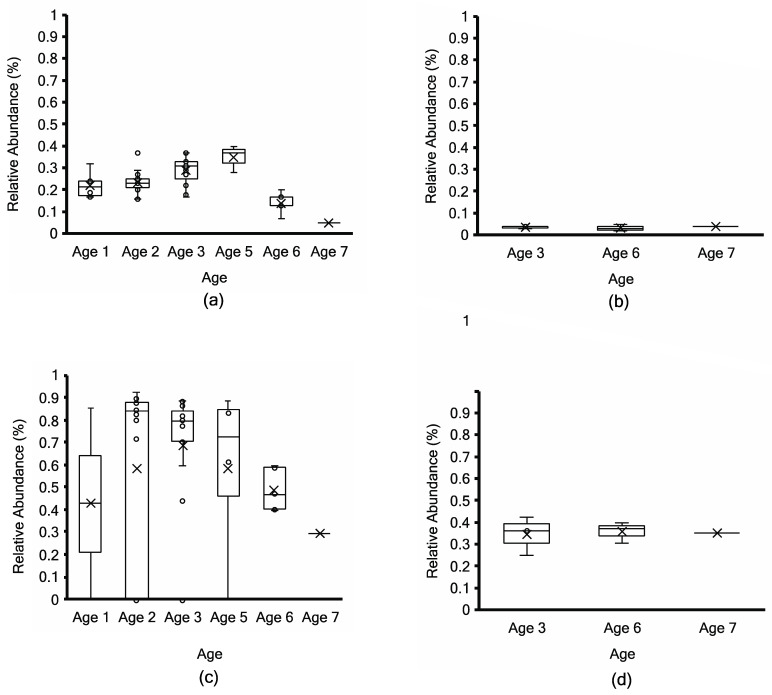
Relative abundance (%) of *Siphoviridae* from Kraken2 taxonomy profiling (top; (**a**,**b**)) and non-assembly-based MG-RAST taxonomy profiling (bottom; (**c**,**d**)) in 51 non-PMA-treated (**a**,**c**) vs. 7 PMA-treated aged Cheddar cheese samples (**b**,**d**). Each box consists of the 75% quartile with a horizontal line representing the median value, accompanied by whiskers showing the 95% confidence interval. The mean value of relative abundance is denoted by “×”, while individual samples are denoted by “o”.

The normalized relative abundance of the two major microbial taxa was compared between Kraken2, non-assembly-based MG-RAST, and the mOTUs2 marker gene cluster-based approach (Figure 5, Figure 6 and Figure 7). To facilitate method comparison and to avoid bias in species and subspecies nomenclature for *Lactococcus*, all reads for *L. cremoris* and *L. lactis* were binned together into *Lactococcus cremoris*/*lactis* (Figure 5). Using Kraken2 taxonomy binning, the relative abundance of *L. lactis* remained inferior to 5% for all trials and cheeses of all ages (Supplementary Tables S3 and S5). By the marker gene method, ref_mOTU_v25_01300 can be attributed to *L. lactis*, and the abundance remained below 0.2% in all samples, while ref_mOTU_v25_01301 designates *L. cremoris* (abundance over 99% by mOTUs2 analysis). *L. paracasei* and *L. casei* taxa were also binned together to avoid bias in nomenclature for comparing methods. Reads classifying as *L. casei* remained below 0.2% of *Lacticaseibacillus* spp. in all samples (Supplementary Figure S4, Tables S4 and S6).

Samples at Age 3 are widely distributed (those denoted by stars in Figure 3) due to the range of *L. lactis* abundance between 87% and 20% using MG-RAST and 96 to 20% using mOTUs2 (Figure 5c,e). Both approaches show a consistent decrease with age progression, while *L. paracasei*/*casei* relative abundance increased over ripening (Figure 6), mainly due to *L. paracasei* (Supplementary Figure S4). The *L. cremoris*/*lactis* relative abundance in the seven PMA-treated samples was less than 2.5% using Kraken2, MG-RAST and mOTUs analyses (Figure 5b,d,f) across the age groups. This highlights that PMA treatment eliminated permeable cells of *L. cremoris*/*lactis*, leaving a minor proportion of intact live cells from 7 m onward. Age 3 was more heterogeneous in *L. cremoris*/*lactis* relative abundance than Age 6. Overall, for *L. paracasei*/*casei* (Figure 6), PMA-treated samples contained a higher relative abundance than non-PMA-treated samples across Ages 3, 6, and 7. The difference between non-PMA and PMA samples across age groups is consistent among the three approaches (Kraken2, MG-RAST non-assembly-based and the mOTU hybrid analyses; Figure 6). The increase in the relative abundance of *L. paracasei*/*casei* over ripening age is consistent between the Kraken2 and mOTUs2 hybrid approaches (Figure 6a,b,e,f), while the MG-RAST approach shows a higher final level (60–90%) at Ages 6 and 7 (Figure 6c,d). Trial 2 cheeses contained the highest overall proportion of *L. paracasei* and the lowest proportion of *L. cremoris* (Supplementary Tables S5 and S6).

Among the remaining *Lactobacillaceae* increasing to 81% abundance at Age 7 (Supplementary Table S4), *Secundilactobacillus* was detected in cheeses at Age 5 (5.7%) and Age 7 (19% of *Lactobacillaceae* reads). *Latilactobacillus* attained a maximum of 8.9% at Age 6, then decreased to under 3% at Age 7. The *Paucilactobacillus* genus remained very low in abundance (at 0.01% or below), then increased to almost 2% of the *Lactobacillaceae* reads at Age 7. The *Levilactobacillus* and *Limosilactobacillus* genera showed a similar profile, remaining low (below 1%) up until Age 6, then increasing to a maximum of 2.8% at Age 7. *Lactiplantibacillus* remained below 2%, and *Pediococcus* remained below 3% throughout aging.

*Siphoviridae* had a low relative abundance of less than 1% (Figure 7) using non-assembly-based taxonomy profiling in fifty-one non-PMA samples and seven PMA samples of ripened cheeses across age groups. Non-PMA-treated samples showed a slightly higher relative abundance of *Siphoviridae* compared to PMA samples. Using MG-RAST, *Ascomycota* were detected with less than 0.07% relative abundance (Supplementary Figure S10), which was below the screening threshold in all samples.

### 3.4. Non-Assembly-Based SNV Frequency Analysis

**Figure 8:**
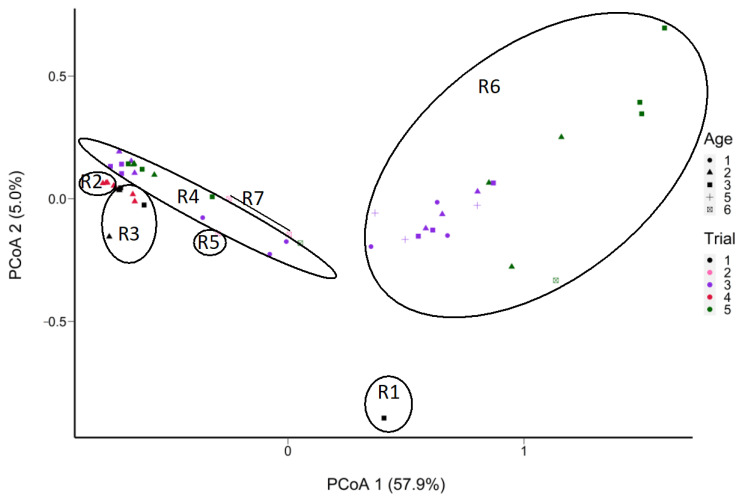
PCoA plot of the Manhattan distance between the cheese samples in terms of the frequencies of SNVs of *L. cremoris* (52 out of 58 samples containing *L. cremoris* reads; absent in 6 samples) determined from non-assembled reads obtained from shotgun sequencing of DNA extracted from aged Cheddar cheeses (5 trials aged to 32 months). Samples S8 and S10 were removed due to insufficient coverage of *L. cremoris.* Sample grouping is outlined based on 7 starters, with ellipses delineating the starters (from R1 to R7).

The starter was the main factor in grouping SNVs of *L. cremoris* in the ripened cheese samples (Figure 8 and Supplementary Figure S5). There were 24 SNVs of *L. cremoris* that varied in frequency according to the starter. Throughout ripening, cheese samples made with starter R6 showed a change in frequency of nine SNVs of *L. cremoris* compared to the other cheese samples and showed separation by trial as well as by age. Starter R6 could be differentiated by the dominance of few SNVs, while the other starters contained higher frequencies of a greater diversity of SNVs (Supplementary Figure S6). Similar trends were observed with samples S1 to S6 in Trial 1, where R1 and R3 starters were not grouped together (Supplementary Figure S5). Based on hierarchical clustering, the SNVs of *L. cremoris* grouped the samples from the same starter at successive ages (Supplementary Figure S6). Moreover, at Age 3, the frequency of one SNV (COG0215–166) belonging to the *L. cremoris* taxon (Supplementary Figure S6) seems to cause sample separation (Figure 8).

**Figure 9:**
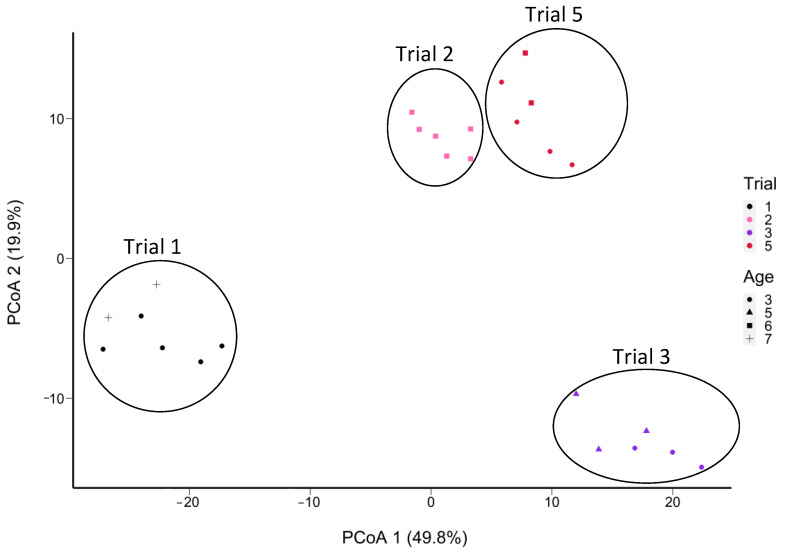
PCoA plot of the Manhattan distance between the cheese samples in terms of the frequencies of SNVs of *L. paracasei*/*casei* (in 28 out of 58 samples total; absent in 30 samples) determined from non-assembled reads obtained from shotgun sequencing of DNA extracted from aged Cheddar cheeses (5 trials aged to 32 months). Samples S6, S32, and S42 were removed due to insufficient coverage of *Lacticaseibacillus*. *L. casei* remained below 1% of the abundance of this taxon. The sample grouping is outlined based on the trial number.

Out of a total of 135 SNVs attributed to *L. paracasei*/*casei*, 73 (64%) were from *L. paracasei* and 62 (46%) were from *L. casei,* while none were attributed to *L. rhamnosus* (Supplementary Tables S7–S16). These SNVs were grouped by trial (Figure 9), regardless of age class. Cheeses from trials 2 and 5 were more similar in their SNV profiles (Figure 9) than cheeses from the three other trials. Moreover, PMA-treated samples (S7, S8, S9) were grouped with their non-PMA counterparts (S4, S5, S6) of the same cheese at the same age (Supplementary Figure S8). The grouping is similar for the samples S13 and S16, as well as S12 to S17. Based on ripening age, a group of 12 SNVs were highly frequent at Ages 3 and 7, but not at the ages in between (Supplementary Figure S9).

## 4. Discussion

The challenge of profiling and characterizing the microbial community during Cheddar cheese ripening is linked to the many sources of variation, such as the initial milk, heat treatment, starter used, aging parameters, quality of samples, and contamination. However, profiling these microorganisms and their potential functional categories is essential to understanding species interactions and how they can influence product characteristics such as flavour. DNA yield could be a limitation in applying molecular methods to aged cheeses due to the fragility of microbial cells during the pelleting and extraction procedures. Sufficient quantity and quality of DNA were obtained from cheeses older than 12 m by increasing the number of cell pellets harvested from 10 g to 60 g of cheese, then combining the extracts for DNA binding and elution without exceeding the binding capacity of the column. One of the main purposes of this study was to establish the diversity of the bacterial communities below the species level and associate their progression during the Cheddar cheese ripening process. Shotgun metagenomics has several advantages over other techniques used to analyze the microbial community in cheese. It allows for the detection of all microorganisms present, including those that may be difficult to culture, and provides information on the functional potential of the microbiota. Additionally, the technique is untargeted and thus does not rely on prior knowledge of the microorganisms present. However, bias may be introduced when analyzing shotgun metagenomics sequences due to mapping the reads to the content of the reference databases. We combined three metagenomic data analysis approaches: an assembly-based strategy using BV-BRC (3.28.21), a non-assembly-based approach using MG-RAST, and a hybrid approach using marker genes with mOTUs2. Each of those approaches contributed to inferring complementary characteristics from fifty-one non-PMA-treated and seven PMA-treated cheese samples.

Assembly-based metagenomics analysis. A key computational challenge in metagenomics is the binning of assembled contigs into draft genomes. The high abundance of repeat regions, the presence of DNA from low-abundance populations in the sample, the inherent noisiness caused by the presence of multiple species in a single sample, and the lack of good reference genomes for unculturable populations make it challenging to produce full-length genomes from metagenomic reads. Many of these problems affect metagenomic assembly, e.g., through the assembly of chimeric contigs, and then propagate these problems into the subsequent binning step. A Metagenome-Assembled Genome (MAG) is a single-taxon assembly based on one or more binned metagenomes that have been asserted to be a close representation of an actual individual genome (that could match an already existing isolate or represent a novel isolate). A MAG shows a microbial genome as a group of sequences from a genome assembly sharing similar characteristics. It enables us to identify novel species and understand their potential functions in a dynamic ecosystem [24].

In this study, cheese samples at Age 3 yielded more MAGs than other ages, and Age 6 cheese samples contained the most diverse communities. Saak et al. [25] determined the MAGs found in washed-rind cheese communities at time points over 13 weeks. Our study identified the distribution of MAGs in Cheddar cheese communities at time points up to 32 months. As observed for washed-rind cheeses, high-quality MAGs across ripening ages of Cheddar cheese contained similar genomes and distinctive genomes depending on the age [25]. Assembly-based approaches take sample reads and match the sequences against a previously assembled genome, leaving unmapped reads to be filtered out. This limits the number of unique species that can be identified and underestimates the true overall abundance of prevalent species.

Comparing non-assembly-based and marker gene metagenomics analysis. The beta diversity of the microbial communities clustered the 58 cheese samples into two groups with some overlap: Ages 1, 2 and then Ages 3 to 7. Cheeses of Age 2 exhibited enough beta diversity to span the distance between the two groups, suggesting a variable ripening rate of these cheeses due mainly to the rate of decline of *Lactococcus* and the concomitant rise in NSLAB. The predicted functions extracted from MG-RAST displayed a separation between PMA-treated and non-PMA-treated samples on the subsystem level. This separation is mainly due to the high proportion of *Lactococcus cremoris* cells that were permeable (eliminated by PMA treatment), while *L. paracasei*/*casei* remained at a higher proportion after PMA treatment, indicating viable cells. The profiles of functions separated the viable and permeable states, although functions associated with stress resistance, carbohydrate and protein degradation were common between the two sets of samples. The comparative analysis showed consistency between MG-RAST and mOTUs2 in characterizing the dominant taxa *Lactococcus cremoris*/*lactis* and *L. paracasei*/*casei* for both non-PMA-treated and PMA-treated samples. The relative abundance of *L. paracasei* increased with age overall but at a variable rate across trials. Previously, 16S rRNA gene amplicon profiling was carried out [15] on a larger set of Cheddar cheese samples compared to this metagenomics study. Both studies show a similar decline for *Lactococcus cremoris*/*lactis.* However, in the previous study, *L. paracasei*/*casei* abundance peaked earlier at a median of 60% (between Ages 4 and 6) and then decreased at Age 7 [15]. In the current study, only one cheese at Age 7 (30–32 m of ripening) was analyzed by shotgun sequencing, leading to a low sample size representing the highest abundance of *L. paracasei*/*casei* (40.5%, which is in the upper range found in the previous study at Age 7). Other *Lactobacillaceae* that increased during later stages of ripening (over 24 m) include those associated with cheese defects, namely *Latilactobacillus* (Age 6), *Paucilactobacillus* and *Secundilactobacillus* (Age 7), in line with the previous study employing 16S rRNA gene amplicon sequencing [15] (Barzideh et al., 2022).

Enrichment of functional categories throughout ripening. The clustering of predicted function categories using PCoA was associated with the relative abundance of the topmost dominant species and demonstrated the separation between PMA and non-PMA groups of samples. While *L. cremoris* was depleted by PMA treatment, *L. paracasei* was enriched. Therefore, the distance between samples by functional categories is mainly due to the relative rates of decline and increase of these taxa. In trials 1 and 2, *Lactococcus cremoris*/*lactis* abundance showed a faster descent rate than in other trials, while trials 3 and 5 maintained higher abundance during ripening. This may be attributed to the rotation of starters between trials, which might vary in rate of lysis. In counterpart, *L. paracasei*/*casei* abundance was higher in trials 1 and 2 (starters R1, R3, R5, R7) than in trials 3 and 5 (Starters R4 and R6) during aging. The most variation in functional profiles seen for Age 3 and 6 cheeses corresponds with the highest diversity in MAG assignments among the age groups. Moreover, this was evident in the profiling results of BV-BRC (3.28.21), MG-RAST, and mOTUs2. Age 3 was a transition point from low relative abundance (0.009) at Age 1 to high relative abundance (0.466) at Age 7 for *L. paracasei*/*casei* and vice versa for *Lactococcus cremoris*/*lactis* that varied from 0.946 at Age 1 to 0.06 at Age 7, with the widest range at Age 3.

The hybrid-based analysis of sequence variation using marker genes showed intraspecies succession with increasing age of the cheese. As expected from starter rotation, the starter is the most important factor differentiating *Lactococcus cremoris*/*lactis* sequence variation among trials. This differential frequency of *L. cremoris*/*lactis* variants may be related to the starter-dependent decline of *Lactococcus,* according to the trial. Starter R6 was associated with low SNV diversity coupled with a high *Lactococcus* proportion at Age 2, suggesting a slower decline rate. Cheeses at Age 2 made with high SNV diversity starters showed beta diversity closer to some Age 3 cheeses with an intermediate relative abundance of *Lactococcus* associated with a higher rate of decline. The *L. paracasei*/*casei* SNV profiles are consistent over aging time within trials. Regardless of age or PMA treatment, however, trial number differentiates the frequency of sequence variants of *L. paracasei*/*casei*, mainly because these NSLAB are entering through the milk. This variation in the subpopulations of *L. paracasei*/*casei* in the incoming milk could result in modifying the aging profile of the cheese.

The dominant good-quality MAGs belonged to *L. cremoris* and *L. paracasei*/*casei*, while MAGs attributed to potential contaminants and spoilage agents such as *Secundilactobacillus*, *Latilactobacillus*, *Paucilactobacillus*, and *Enterobacter* were found at later ripening stages (Age 6). Both MG-RAST and mOTUs2 showed similar profiling results for *Lactococcus cremoris*/*lactis* and *L. paracasei*/*casei,* which corresponds with their dominance using the assembly-based analysis pipeline of BV-BRC (3.28.21). However, since the assembly-based approach depends on MAGs with good bins, the number of reads for low-abundance taxa may limit the quality of the assembly. The non-assembly-based approach adds the ability to profile the abundance of functional categories, adding phages and fungi to bacteria, while the hybrid method contributes to infra-species sequence variation.

The MG-RAST and marker gene-based mOTUs2 hybrid approaches provided similar profiling results for the dominant taxa. Sub-species and non-dominant taxa, such as *Siphoviridae* phages and fungi (*Ascomycota*), with low relative abundance were obtained using the MG-RAST non-assembly-based approach, while they were not detected using the assembly-based approach due to the binning quality, especially for *Ascomycota*. *Siphoviridae* were detected at a low relative abundance of less than 1%, which may be due to the low binning success of prophage sequences, even though 74% of *Lactococcus* MAGs contained prophage sequences. Artisanal cheeses of three types have shown an almost 20% abundance of phages in taxonomic profiling using assembly-based analysis of shotgun sequences [24].

Shotgun metagenomics involves the sequencing of DNA extracted from a sample without any prior knowledge of the community composition and has been used to study the microbial diversity and functional potential of cheese microbiota, including feta cheese [26]. After two months of ripening, the homemade cheeses could be distinguished from industrial cheeses based on the high prevalence of *Lactococcus* in the former, while *Streptococcus thermophilus* and *Lactobacillus delbrueckii* subsp. *bulgaricus* dominated the microbiota of the latter. MAGs for foodborne pathogens were in low abundance. However, only assembly-based sequence analysis was carried out, while yeast and fungal abundances were evaluated by ITS sequencing, not by shotgun sequencing. Thus, the relative abundance of bacteria and fungi relative to each other could not be assessed. In a study of surface-ripened cheeses from 10 countries, the succession of bacteria was not specific to a geographical location but could be reproduced over time (63 days) in an in vitro system [27]. Assembly-based analysis of shotgun sequences has also been carried out on Irish artisanal cheeses obtained from 27 artisanal farm producers or farmer’s markets to show the variability among 55 cheeses (15 soft, 16 semi-hard and 24 hard cheeses) made from unpasteurized or pasteurized sheep, goat or cow’s milk [24]. Out of 32 MAGS, 47 new species were putatively identified that could contribute to taste and colour. Volatile levels could be correlated with the abundance of strains. Five species of phages belonging to the *Siphoviridae* were detected. This data was combined with 107 publicly available cheese metagenomes. Overall, viral signals in 88 MAGs were hypothetically lysogenic, with 74% of *Lactococcus* MAGs and 6% of *Streptococcus* MAGs containing prophages. In our study, we also found that 74% of *Lactococcus* MAGs contained *Siphoviridae* prophage sequences but no hypothetically lysogenic signals.

PMA treatment was very useful to discriminate the viable bacteria surviving HTST processing of milk used for Cheddar cheese-making in a previous study [16]. Thermoduric heterofermentative *Lactobacillus* was detected at low levels in cheeses with slit defects. That study also showed an increase in *Lactobacillus* NSLAB when aging over the course of 120 days. As samples were treated with PMA during DNA extraction in that study, the results reflect a presumably viable population of bacteria. The relative abundance of *Lactobacillus* spp. reached 50% at 120 days, but the values were quite widely spread between 40% and 60% from 30 to 90 days of ripening [16]. In our study, PMA treatment allowed us to estimate the relative abundance of viable *L. paracasei* at 80–90% at 30–32 m of ripening while showing a very low abundance of *L. casei* (under 0.1%).

Washed-rind cheeses made from raw or pasteurized milk were analyzed up to 13 weeks of ripening [25] by 16S rRNA gene amplicon, ITS and metagenomic shotgun sequencing. The results showed that the microbial communities in three batches of raw milk cheese were more diverse and had a higher abundance of non-starter bacteria compared to washed-rind cheeses made with pasteurized milk. Batch-to-batch variation was evidenced, particularly with the presence of *Fusarium* in some but not all batches of pasteurized milk cheese and not in the raw milk cheese. Functional profiling was focused on the *Psychrobacter* genus, differentiating core functions present in all eight MAGs (amino acid metabolism) and comparing isolate genomes with unique functions (phages and transposons). However, functional profiling was not used to differentiate cheeses made from raw milk or pasteurized milk. In a study on Cheddar cheese using 16S rRNA gene amplicon sequencing (genus-level taxonomy from total DNA), the addition of prebiotics (2% inulin or FOS) led to a significantly higher abundance of *Lactobacillaceae* NSLAB after 90 days of ripening, but the level attained was about 15% at this age [28]. Either smoking or soaking Cheddar cheese in beverages (pinot noir wine, hard apple cider or porter beer) affected the succession of bacterial taxa on the surface versus the core of the cheeses over 3 to 6 m of aging [29]. *Lactobacillaceae* were particularly enriched in cheeses soaked with pinot noir and porter, up to 60% in abundance on the surface but only 40% in the core of the cheeses. Again, functions were extrapolated from the genomes associated with the taxa. Functions associated with carbohydrate metabolism were enriched in Cheddar cheeses either smoked or soaked in wine or porter, while functions associated with amino acid metabolism were slightly lower in abundance compared to plain Cheddar cheese aged at the same time [30]. In the current study at a similar age (3–6 m; Age 2), *Lactobacillaceae* composed only 6% of the microbial community, which is lower, while *L. cremoris* relative abundance was still quite high at 82%. Functions associated with stress (DNA repair, acid resistance, starvation, alternate sugar utilization) and amino acid conversion were enriched during ripening, mostly after 18–20 m (Age 5).

Metatranscriptomics is based on sequencing the mRNA produced by live cells, which more directly targets viability compared to PMA treatment of cells before DNA extraction. This challenging approach was used to profile surface-ripened cheeses over 31 days of ripening [31]. The most active species on Day 1, *L. lactis* and *Kluyveromyces lactis*, were replaced on Day 7 by *Geotrichum candidum,* while acid-sensitive ripening bacteria increased only at the end of the 31 days [31]. Functional classification of the metatranscriptome revealed the abundance of lactose metabolism early in ripening, replaced by lactate metabolism from Days 7 to 21, related to deacidification of the curd. After building specific classes for proteases and peptidases, classification of the RNA-Seq data showed that proteolytic and amino acid metabolism functions from *G. candidum* were dominant over the ripening period. Biomarkers were selected by comparative analysis of sampling points to represent glycolysis, protein and peptide degradation, amino acid metabolism, lipolysis and stress [31]. Since then, a variety of cheeses have been analyzed using metatranscriptomics. De Fillipis (2016) demonstrated temperature-driven changes in metabolic activities that were related to the maturation rate of Italian cheeses. Metatranscriptomics of Austrian washed-rind hard cheeses before (30 d) and after (90 d) ripening showed the transition to higher expression of amino acid and fatty acid metabolism by *Brevibacterium* and *Corynebacterium,* leading to the generation of compounds such as 2,3-butanediol and methanethiol [32]. The challenge at this point is the strain-dependent variation within species, for example in the case of *L. casei* in long-ripened cheeses [8]. Whole genome sequencing has been shown to be reliable for documenting strain-specific differences in many lactic acid bacteria, starting with *L. lactis* [33,34], *L. casei* [35], the *Lactobacillus* genus [36], and most recently the complete restructuring of the *Lactobacillaceae* [37]. However, this approach may be biased by the process of obtaining isolates through culturing. Metagenomics shotgun sequencing contributes to bridging the gap between genus-level profiling and WGS to identify the diversity within species, ideally at the strain level. However, the marker gene approach relies on single-copy genes conserved in numerous bacterial species, which may not have enough resolution at the strain level. Rather, single nucleotide variants in conserved marker genes may reflect groups of strains with specific genotypes. The SNV variation found in the current study should be corroborated by whole genome sequencing in the future to determine the actual strain diversity.

In our study, integrating three metagenomics approaches into the analysis provided profiling of taxa and SNVs in fifty-one non-PMA-treated cheese samples and seven PMA samples. Metagenomic binning with BV-BRC (3.28.21) enabled the assembly of MAGs with good quality associated with each ripening age. However, phages and fungi sequences did not pass the binning quality threshold by the assembly-based analysis. The non-assembly-based MG-RAST enabled the detection of phage and fungal reads as well as inferring predicted function categories. The function profiles could discriminate cheese age as well as PMA treatment, which shows the difference between total bacterial DNA and DNA from presumably live cells (PMA-treated). The added value of the hybrid approach using mOTUs2 based on marker gene clusters provided infra-species-level variation by determining SNVs of the dominant taxa.

## 5. Conclusions and Perspectives

This metagenomics study differentiated the profiles of predicted function categories according to PMA or non-PMA treatment as well as age and taxa in ripened Cheddar cheese samples. A collection of fifty-one non-PMA and seven PMA samples gathered from five trials, each using two starters, were analyzed across 32 months of aging. The most dominant species were *Lactococcus cremoris* and *Lacticaseibacillus paracasei*. The sequence diversity of *Lactococcus cremoris* discriminated the starters, while trial (milk origin) was the main factor affecting the sequence diversity of *Lacticaseibacillus paracasei*/*casei*. Previously, 16S rRNA gene amplicon sequencing was limited to genus-level taxonomic resolution, requiring culturomic approaches to further differentiate at the strain level. Assembling genomes from metagenomic data (MAGs) has further refined the ability to identify within-species sequence variants in ripening cheeses. Shotgun metagenomics was able to distinguish among starters according to the overall frequency of certain SNVs, which were related to the rate of decline of *Lactococcus* at Age 2. One starter (R6) had lower SNV diversity coupled with a slower decline, whereas most starters maintained a higher diversity of SNVs across trials and age groups. The stability of SNV profiles of *L. paracasei*/*casei* within trials suggests that initial subpopulations from milk survive over time. The non-assembly-based analytical method used in the current study to profile SNVs provides insight into how the subpopulation of *Lacticaseibacillus* can vary among trials, which has implications for eventually predicting the range of variation in flavour development over time. Further investigation of *L. paracasei* isolates is needed to correlate the genotype with flavour development in these cheeses. Infra-species sequence variants of dominant taxa and significantly enriched functions helped further understand interactions between subpopulations in cheese throughout ripening. This can help relate genotype to phenotype as well as identify potential strains that could be used as adjunct starters to target specific flavour profiles. In the future, shotgun metagenomics data could be integrated with volatile profiles to associate specific microbe variants with the development of compounds contributing to flavour. Precise and reliable predictive markers could help verify product authenticity, particularly with regard to cheese ripening stages.

## Figures and Tables

**Table 1:** Distribution of Cheddar cheese samples for which sufficient DNA was extracted for shotgun sequencing, showing the total number of non-PMA-treated samples at each ripening age and the corresponding number of trials.

Age (Months)							
Number of Trials and Samples	Age 1 (0–1 m)	Age 2 (3–6 m)	Age 3 (7–10 m)	Age 5 (18–20 m)	Age 6 (24 m)	Age 7 (30–32 m)	Total
Number of trials	1	4	3	1	2	1	5
Number of samples	6	21	15	3	5	1	51

## Data Availability

The datasets for this study are available in the Agri-Environmental Research Data Repository of the University of Guelph at https://doi:10.5683/SP3/KFHWLK.

## Supplementary Materials

The following supporting information can be downloaded at: https://www.mdpi.com/article/10.3390/microorganisms11082052/s1, Figure S1: Assembly and characterization of cheese MAGs and the quality of MAG binning as determined by BV-BRC (3.28.21); Figure S2: Total number of MAGs (**a**) and assigned genera (**b**); Figure S3: Shepard plot of Multivariate nonlinear multidimensional scaling (NMDS) analysis of cheese shotgun samples across all age groups; Figure S4: Taxonomic profiling by Kraken2 of the *L. cremoris* and *L. lactis* populations (**a**,**b**) and *L. paracasei* and *L. casei* (**c**,**d**); Figure S5: SNVs of *L. cremoris* ordered by trial, and each trial had two starters; Figure S6: Heatmap of the frequencies of *Lactococcus cremoris* SNVs obtained from non-assembled reads ordered by starter, trial number and age of the cheese; Figure S7: Heatmap of the frequencies of *Lactococcus cremoris* SNVs obtained from non-assembled reads ordered, first by age of the cheese, then by trial number; Figure S8: Heatmap of the frequency of SNVs of *Lacticaseibacillus paracasei*/*casei* (73 from *L. paracasei* and 62 from *L. casei*) identified from non-assembled reads ordered first by trial number and then age of cheese; Figure S9: Heatmap of the frequency of SNVs of *Lacticaseibacillus paracasei*/*casei* (73 from *L. paracasei* and 62 from *L. casei*) identified from non-assembled reads ordered first by age and then by trial number; Figure S10: *Ascomycota* taxa profiling using the non-assembly-based MG-RAST approach; Table S1: PMA-treated sample sequencing breakdown analysis statistics; Table S2: Non-PMA sample sequencing breakdown analysis statistics; Table S3: Relative abundance (%) by Kraken2 of *Lactococcus*, *L. cremoris*, *L. lactis* and unclassified *Lactococcus* across age groups; Table S4: Relative abundance (%) by Kraken2 of *Lacticaseibacillus*, *L. paracasei*, *L. casei* and unclassified *L. paracasei*/*casei* across age groups; Table S5: Relative abundance (%) by Kraken2 of *Lactococcus*, *L. cremoris*, *L. lactis* and unclassified *Lactococcus* across trials; Table S6: Relative abundance (%) by Kraken2 of *Lacticaseibacillus*, *L. paracasei*, *L. casei* and unclassified *L. paracasei*/*casei* across trials; Tables S7–S16: Species attribution of SNVs by COG.
