# Supplementary material for: Taxonomy, Sequence Variance and Functional Profiling of the Microbial Community of Long-Ripened Cheddar Cheese Using Shotgun Metagenomics
Source: Microorganisms. 2023 Aug 10;11(8):2052. doi: 10.3390/microorganisms11082052 (PMC10458550; doi:10.3390/microorganisms11082052)
Supplement: Supplementary file 1 [file microorganisms-11-02052-s001.zip › microorganisms-2449686-supplementary.pdf]

Supplementary Material for Mohamed et al. (2023).

Table S1. PMA samples sequencing breakdown analysis statistics; showing sequence length, mean GC percent; before and after QC; in addition to sequences containing annotated proteins and predicted rRNA and their percentiles, besides α-diversity per each sample.

			Before QC		Post QC					
**Age**	**Trial**	**S#**	**Mean Sequence Length (bp)**	**Mean GC (%)**	**Mean Sequence Length (bp)**	**Mean GC (%)**	**Number of sequences with predicted proteins (%)**	**Number with predicted rRNA features (%)**	**Sequence Passed QC (%)**	**α-diversity**
3	1	7	187 ± 33	45 ± 5	191 ± 33	45 ± 5	2,447,789 (95.61%)	21,168 (0.83%)	76.10	5
3	1	8	187 ± 33	45 ± 6	191 ± 33	45 ± 6	2,731,154 (95.46%)	25,525 (0.89%)	74.27	7
3	1	9	189 ± 33	45 ± 6	193 ± 33	45 ± 6	2,263,862 (95.06%)	20,867 (0.88%)	78.40	7
7	1	11	188 ± 33	45 ± 6	195 ± 32	45 ± 6	3,555,055 (95.64%)	30,365 (0.82%)	65.16	6
6	2	15	186 ± 33	42 ± 6	189 ± 33	42 ± 6	2,972,375 (94.13%)	26,508 (0.84%)	81.45	23
6	2	16	187 ± 33	39 ± 6	191 ± 33	40 ± 6	2,469,208 (93.98%)	23,489 (0.89%)	75.46	38
6	2	17	186 ± 33	43 ± 7	188 ± 33	43 ± 7	2,761,798 (93.03%)	25,052 (0.84%)	86.21	22

Table S2. Non-PMA samples sequencing breakdown analysis statistics; showing sequence length, mean GC percent; before and after QC; in addition to sequences containing annotated proteins and predicted rRNA and their percentiles, besides α-diversity per each sample.

			Before QC		Post QC					
**Age**	**Trial**	**S#**	**Mean Sequence Length (bp)**	**Mean GC (%)**	**Mean Sequence Length (bp)**	**Mean GC (%)**	**Number of sequences with predicted proteins (%)**	**Number with predicted rRNA features (%)**	**Sequence Passed QC (%)**	**α-diversity**
6	5	58	185 ± 33	40 ± 7	189 ± 33	41 ± 7	2,988,458 (93.82%)	28,855 (0.91%)	75.63	25
6	5	57	185 ± 33	40 ± 7	192 ± 33	41 ± 7	6,591,642 (92.94%)	67,007 (0.94%)	63.05	25
3	5	56	188 ± 33	36 ± 6	194 ± 33	36 ± 7	2,235,717 (95.61%)	18,361 (0.79%)	58.3	8
3	5	55	186 ± 33	35 ± 6	194 ± 33	35 ± 6	2,225,087 (95.91%)	18,818 (0.81%)	57.49	7
3	5	54	187 ± 33	39 ± 8	191 ± 33	40 ± 8	2,591,740 (94.58%)	20,631 (0.75%)	71.16	9
3	5	53	186 ± 33	36 ± 7	191 ± 33	38 ± 7	2,807,647 (93.25%)	23,464 (0.78%)	62.03	15
3	5	52	183 ± 33	37 ± 7	189 ± 34	38 ± 7	2,793,452 (94.41%)	21,624 (0.73%)	60.22	10
3	5	51	187 ± 33	35 ± 6	194 ± 33	35 ± 6	2,092,687 (95.93%)	18,901 (0.87%)	58.79	6
2	5	50	241 ± 5	33 ± 7	241 ± 6	33 ± 7	32,722 (86.86%)	426 (1.13%)	50.87	4
2	5	49	187 ± 33	35 ± 6	194 ± 33	35 ± 6	2,241,549 (95.86%)	23,970 (1.03%)	61.93	6
2	5	48	184 ± 33	35 ± 6	191 ± 33	35 ± 6	2,480,307 (96.19%)	25,459 (0.99%)	58.67	5
2	5	47	185 ± 33	35 ± 6	192 ± 33	35 ± 6	2,272,143 (95.83%)	22,704 (0.96%)	58.49	5
2	5	46	187 ± 33	35 ± 6	194 ± 33	35 ± 6	2,158,402 (95.93%)	22,327 (0.99%)	58.97	5

2	5	45	185 ± 33	35 ± 6	197 ± 33	35 ± 6	3,636,655 (95.49%)	36,035 (0.95%)	43.4	5
2	4	44	185 ± 33	35 ± 6	192 ± 33	35 ± 6	2,197,777 (95.53%)	23,471 (1.02%)	61.1	6
2	4	43	186 ± 33	35 ± 6	193 ± 33	35 ± 6	2,243,083 (95.96%)	24,402 (1.04%)	61.11	5
2	4	42	185 ± 33	36 ± 6	191 ± 33	36 ± 6	2,361,497 (95.02%)	25,523 (1.03%)	64.49	9
2	4	41	186 ± 33	36 ± 6	191 ± 33	36 ± 6	2,408,031 (94.94%)	27,763 (1.09%)	68.19	11
2	4	40	184 ± 33	36 ± 6	190 ± 33	36 ± 6	2,432,981 (95.20%)	26,182 (1.02%)	65.57	8
2	4	39	186 ± 33	35 ± 6	192 ± 33	35 ± 6	2,184,288 (95.76%)	22,374 (0.98%)	60.74	5
5	3	38	184 ± 33	36 ± 7	191 ± 33	37 ± 7	2,193,189 (94.99%)	19,989 (0.87%)	58.1	9
5	3	37	186 ± 33	38 ± 8	191 ± 33	39 ± 8	2,578,281 (94.60%)	23,454 (0.86%)	64.44	11
5	3	36	186 ± 33	39 ± 9	189 ± 33	41 ± 9	2,740,067 (94.10%)	28,077 (0.96%)	72.45	20
3	3	35	183 ± 33	35 ± 6	189 ± 34	35 ± 6	2,282,138 (95.99%)	23,845 (1.00%)	63.63	5
3	3	34	184 ± 33	35 ± 6	191 ± 33	36 ± 6	2,537,754 (95.67%)	25,629 (0.97%)	59.53	6
3	3	33	211 ± 27	35 ± 6	212 ± 27	35 ± 6	318,268 (94.69%)	2,879 (0.86%)	90.27	5
3	3	32	185 ± 33	36 ± 6	192 ± 34	36 ± 6	2,101,623 (95.38%)	19,782 (0.90%)	60.87	6
3	3	31	182 ± 33	36 ± 7	189 ± 34	36 ± 7	2,470,194 (94.75%)	22,805 (0.87%)	59.94	8
3	3	30	186 ± 33	36 ± 7	192 ± 33	37 ± 7	2,221,486 (94.20%)	20,717 (0.88%)	62.97	10
2	3	29	210 ± 28	35 ± 6	212 ± 27	35 ± 6	255,135 (94.21%)	2,279 (0.84%)	90.18	5
2	3	28	185 ± 33	35 ± 6	192 ± 33	35 ± 6	2,030,342 (96.24%)	18,866 (0.89%)	62.17	5
2	3	27	210 ± 28	35 ± 6	211 ± 28	35 ± 6	230,121 (93.66%)	2,042 (0.83%)	91.47	6
2	3	26	210 ± 28	35 ± 6	212 ± 27	35 ± 6	314,211 (94.01%)	2,824 (0.84%)	88.91	7
2	3	25	208 ± 30	35 ± 6	209 ± 29	35 ± 6	168,225 (92.67%)	1,587 (0.87%)	90.72	7
2	3	24	209 ± 29	35 ± 6	211 ± 28	35 ± 6	194,301 (92.36%)	1,831 (0.87%)	90.93	8
1	3	23	207 ± 30	35 ± 6	209 ± 29	35 ± 6	159,788 (93.02%)	1,484 (0.86%)	92.57	5
1	3	22	206 ± 31	35 ± 6	207 ± 30	35 ± 6	133,671 (92.47%)	1,178 (0.81%)	92.83	4
1	3	21	211 ± 28	35 ± 6	212 ± 27	35 ± 6	308,800 (94.55%)	2,781 (0.85%)	90.96	4
1	3	20	208 ± 29	35 ± 6	210 ± 28	35 ± 6	236,139 (93.67%)	2,099 (0.83%)	88.9	5
1	3	19	206 ± 30	35 ± 6	207 ± 30	35 ± 6	130,339 (92.35%)	1,189 (0.84%)	91.57	4
1	3	18	211 ± 27	35 ± 6	213 ± 26	35 ± 6	540,999 (95.21%)	4,955 (0.87%)	85.07	5
6	2	14	185 ± 33	40 ± 7	187 ± 33	41 ± 7	3,088,482 (92.50%)	26,639 (0.80%)	82.89	29
6	2	13	184 ± 33	39 ± 6	188 ± 33	39 ± 6	2,971,855 (93.26%)	29,014 (0.91%)	78.77	42
6	2	12	184 ± 33	39 ± 7	187 ± 33	40 ± 7	3,012,266 (93.19%)	25,133 (0.78%)	81.42	30
7	1	10	186 ± 33	42 ± 7	188 ± 33	42 ± 7	2,349,784 (93.06%)	17,383 (0.69%)	83.09	23
3	1	6	187 ± 33	36 ± 6	193 ± 33	36 ± 6	2,144,401 (95.04%)	21,981 (0.97%)	64.3	8
3	1	5	190 ± 33	42 ± 8	192 ± 33	42 ± 7	2,265,257 (93.78%	21,502 (0.89%)	80.08	10
3	1	4	186 ± 33	38 ± 8	190 ± 33	39 ± 7	2,616,985 (94.76%)	25,661 (0.93%)	70.65	10
2	1	3	186 ± 33	35 ± 6	193 ± 33	36 ± 6	2,411,369 (95.44%)	24,834 (0.98%)	61.14	7
2	1	2	186 ± 33	35 ± 6	193 ± 33	35 ± 6	2,156,235 (96.03%)	23,314 (1.04%)	61.9	4
2	1	1	186 ± 33	35 ± 6	193 ± 33	35 ± 6	2,327,704 (95.97%)	25,276 (1.04%)	62.29	5

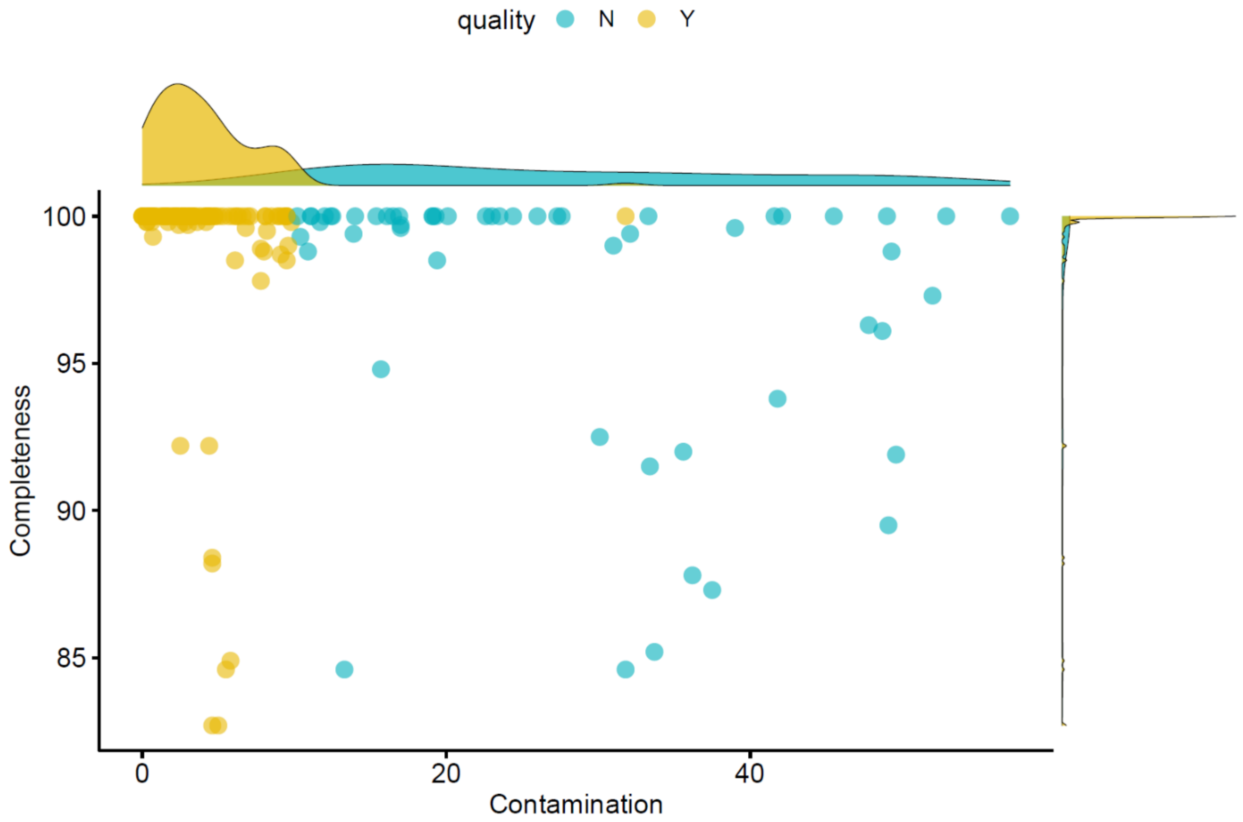

Figure S1. Assembly and characterization of cheese MAGs, the quality of MAGs binning as determined by BV-BRC (3.28.21) MAGs binned with good quality (n=110) out of total (n=165) are shown in yellow dots, while MAGs did not pass the quality are with cyan dots. Quality=Y means that these bins met the quality criteria of completeness >= 80%, fine consistency >= 87%, contamination <= 10%, and have a single PheS protein of reasonable size.

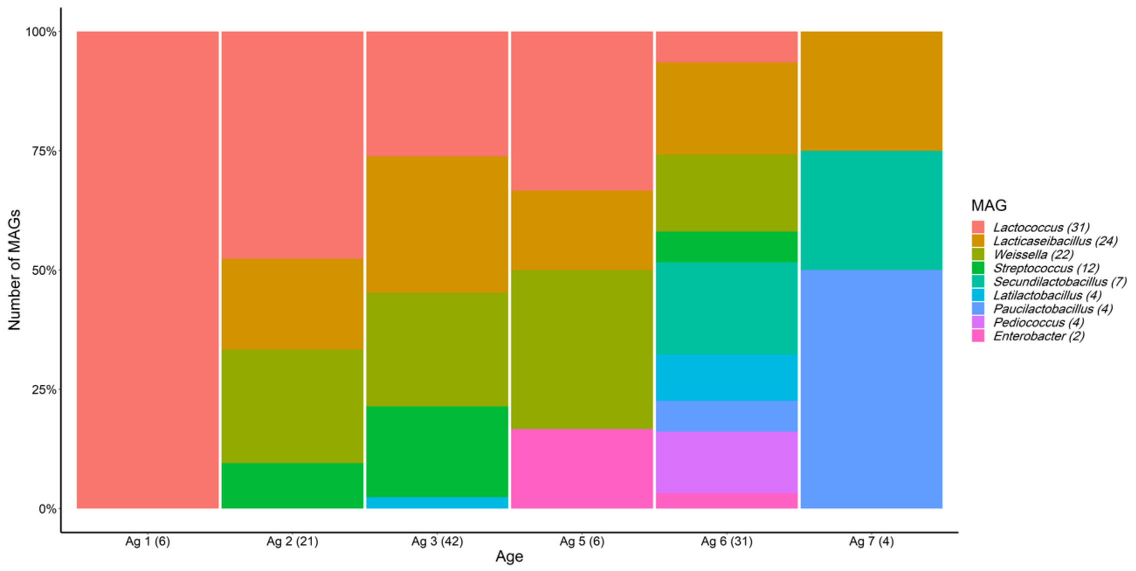

(a)

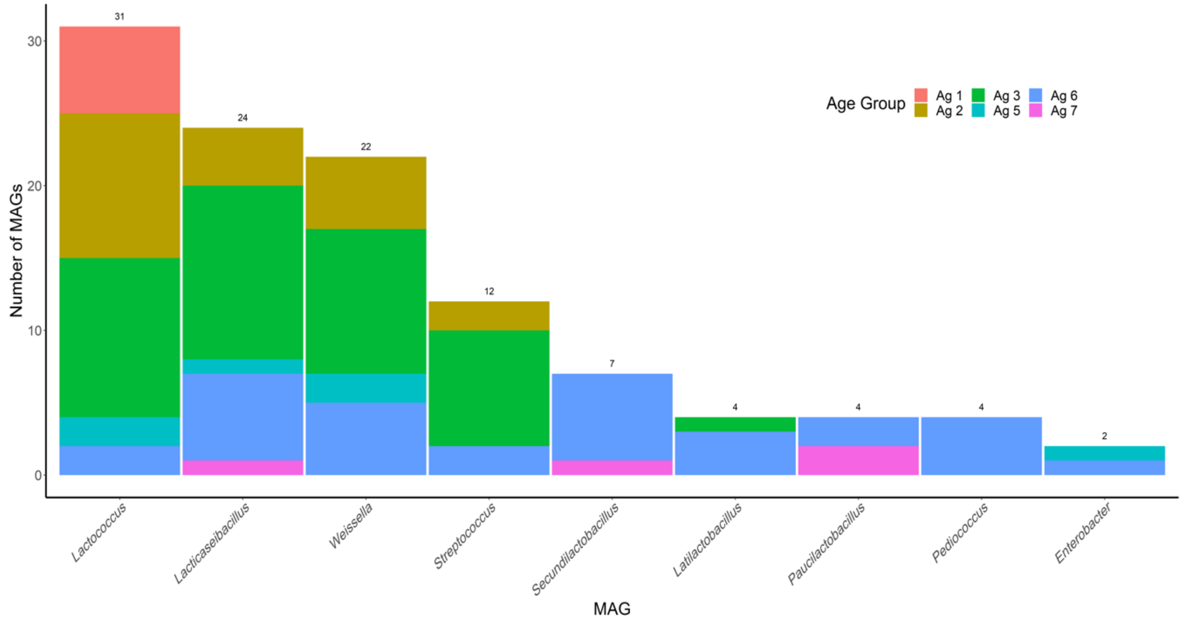

(b)

Figure S2. Total number of MAGs with good quality detected using metagenomic binning. (a) Distribution of genera where MAGs were assigned across age groups (Y-axis). (b) The genera to which MAGs were assigned are shown on the X-axis for each age group, and the Y-axis shows the total number of good quality MAGs of each genus.

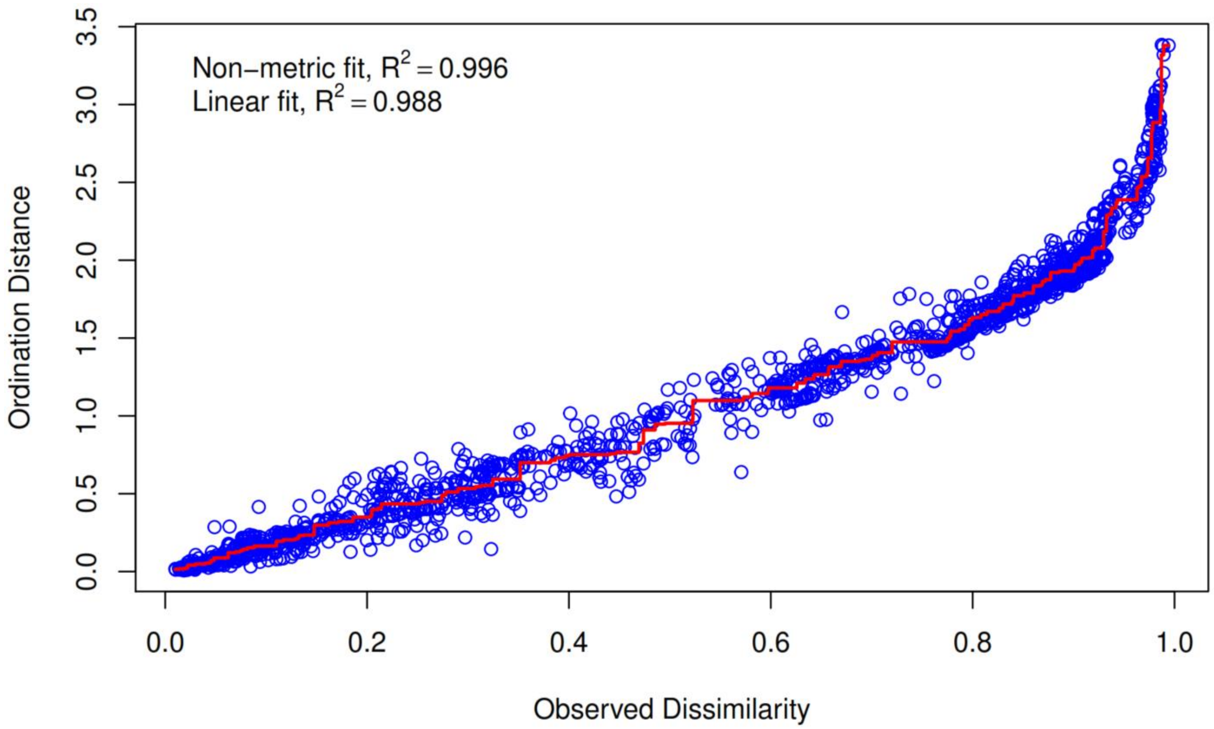

Figure S3. Shepard plot of Multivariate nonlinear multidimensional scaling (NMDS) analysis of cheese shotgun samples across all age groups.

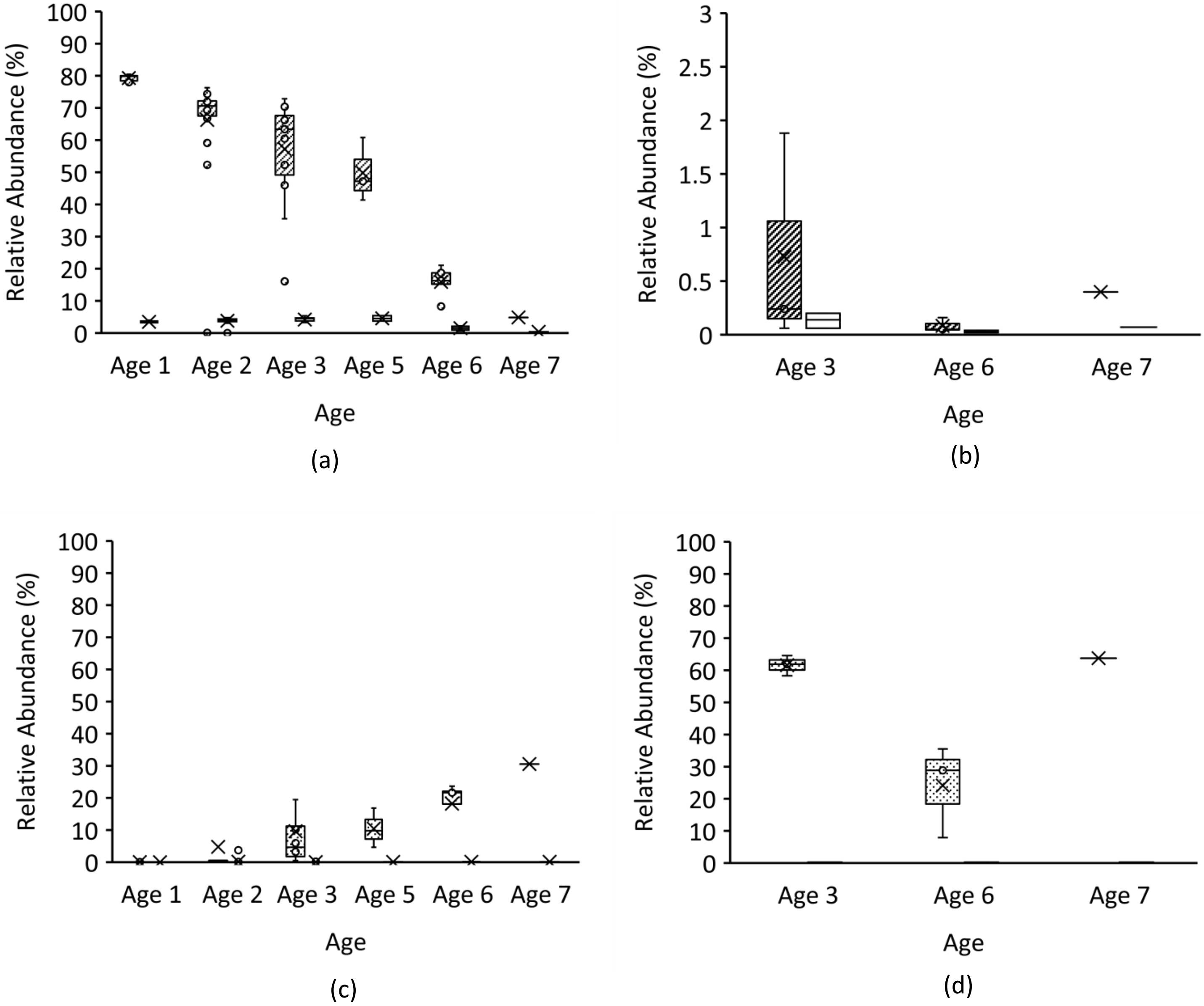

Figure S4. Taxonomic profiling by Kraken2 of *L. cremoris* (stripped box) and *L. lactis* (blank box) population (a,b) and *L. paracasei* (dotted box) and *L. casei* (blank box close to x-axis) population (c,d) in 51 non-PMA treated cheese samples (left) and 7 PMA treated cheese samples (right).

Table S3. Relative abundance (%) by Kraken2 of total *Lactococcus lactis, L. cremoris*, *L. lactis* and unclassified *Lactococcus lactis* of 51 non-PMA-treated aged cheese samples across age groups.

Taxonomy	Relative Abundance (%)					
	Age 1 (n = 6)	Age 2 (n = 21)	Age 3 (n = 15)	Age 5 (n = 3)	Age 6 (n = 5)	Age 7 (n = 1)
Lactococcus	93.0 ± 1.03	81.9 ± 5.51	73.7 ± 18.7	66.9 ± 13.2	21.7 ± 7.12	6.4 ± 0.0
L. cremoris	79.3 ± 1.20	66.3 ± 5.56	57.2 ± 15.6	49.8 ± 9.98	15.9 ± 4.82	4.9 ± 0.0
L. lactis	3.5 ± 0.29	3.82 ± 0.46	4.2 ± 0.99	4.6 ± 0.83	1.5 ± 0.62	0.4 ± 0.0
unclassified *Lactococcus*	10.3 ± 0.79	11.8 ± 1.09	12.2 ± 2.71	12.6 ± 2.51	4.3 ± 1.73	1.2 ± 0.0

Table S4. Relative abundance (%) by Kraken2 of total *Lacticaseibacillus, L. paracasei*, *L. casei* and unclassified *Lacticaseibacillus paracasei/casei* of 51 non-PMA-treated aged cheese samples across age groups.

Taxonomy	Relative Abundance (%)					
	Age 1 (n = 6)	Age 2 (n = 21)	Age 3 (n = 15)	Age 5 (n = 3)	Age 6 (n = 5)	Age 7 (n = 1)
Lacticaseibacillus	0.0 ± 0.0	5.9 ± 3.63	12.6 ± 16.6	13.7 ± 7.99	23.9 ± 9.54	40.5 ± 0.0
L. paracasei	0.0 ± 0.0	4.8 ± 2.76	9.6 ± 12.7	10.4 ± 6.12	18.2 ± 7.37	30.6 ± 0.0
L. casei	0.0 ± 0.0	0.2 ± 0.015	0.1 ± 0.06	0.1 ± 0.04	0.1 ± 0.05	0.1 ± 0.0
unclassified *Lacticaseibacillus paracasei/casei*	0.0 ± 0.0	0.9 ± 0.86	2.9 ± 3.87	3.2 ± 1.83	5.6 ± 2.16	9.8 ± 0.0

Table S5. Relative abundance (%) by Kraken2 of total *Lactococcus lactis, L. cremoris*, *L. lactis* and unclassified *Lactococcus lactis* of 51 non-PMA-treated aged cheese samples according to trial.

Taxonomy	Relative Abundance (%)				
	Trial 1 (n = 7)	Trial 2 (n = 3)	Trial 3 (n = 21)	Trial 4 (n = 6)	Trial 5 (n = 14)
Lactococcus	62.1 ± 34.8	17.6 ± 5.97	80.7 ± 20.8	82.0 ± 8.41	73.1 ± 22.7
L. cremoris	50.2 ± 29.0	13.3 ± 4.33	65.8 ± 18.2	65.7 ± 8.44	56.6 ± 19.0
L. lactis	2.88 ± 1.43	1.11 ± 0.42	3.83 ± 1.02	3.95 ± 0.45	4.16 ± 1.07
unclassified *Lactococcus*	9.03 ± 4.55	3.26 ± 1.28	11.1 ± 2.93	12.3 ± 0.89	12.3 ± 3.04

Table S6. Relative abundance (%) by Kraken2 of total *Lacticaseibacillus, L. paracasei*, *L. casei* and unclassified *Lacticaseibacillus paracasei/casei* of 51 non-PMA-treated aged cheese samples according to trial.

Taxonomy	Relative Abundance (%)				
	Trial 1 (n = 7)	Trial 2 (n = 3)	Trial 3 (n = 21)	Trial 4 (n = 6)	Trial 5 (n = 14)
Lacticaseibacillus	18.9 ± 23.5	20.3 ± 11.5	7.49 ± 20.1	4.64 ± 6.05	9.70 ± 13.4
L. paracasei	14.4 ± 17.9	15.2 ± 8.63	6.01 ± 16.5	3.52 ± 4.59	7.47 ± 10.4
L. casei	0.06 ± 0.06	0.11 ± 0.06	0.20 ± 0.81	0.02 ± 0.03	0.06 ± 0.06
unclassified *Lacticaseibacillus paracasei/casei*	4.52 ± 5.55	4.96 ± 2.80	1.28 ± 2.75	1.09 ± 1.43	2.17 ± 3.01

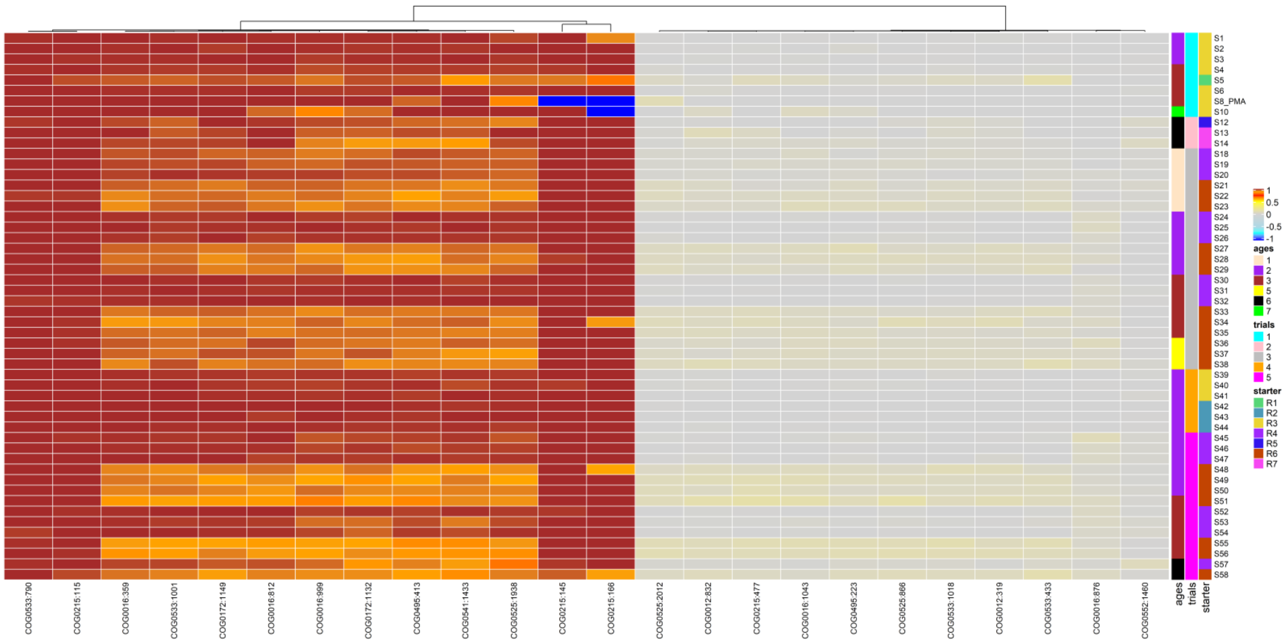

Figure S5. SNVs of *Lactococcus cremoris* ordered by trial (i.e., same cheese), each trial had two starters as shown on the right legend.

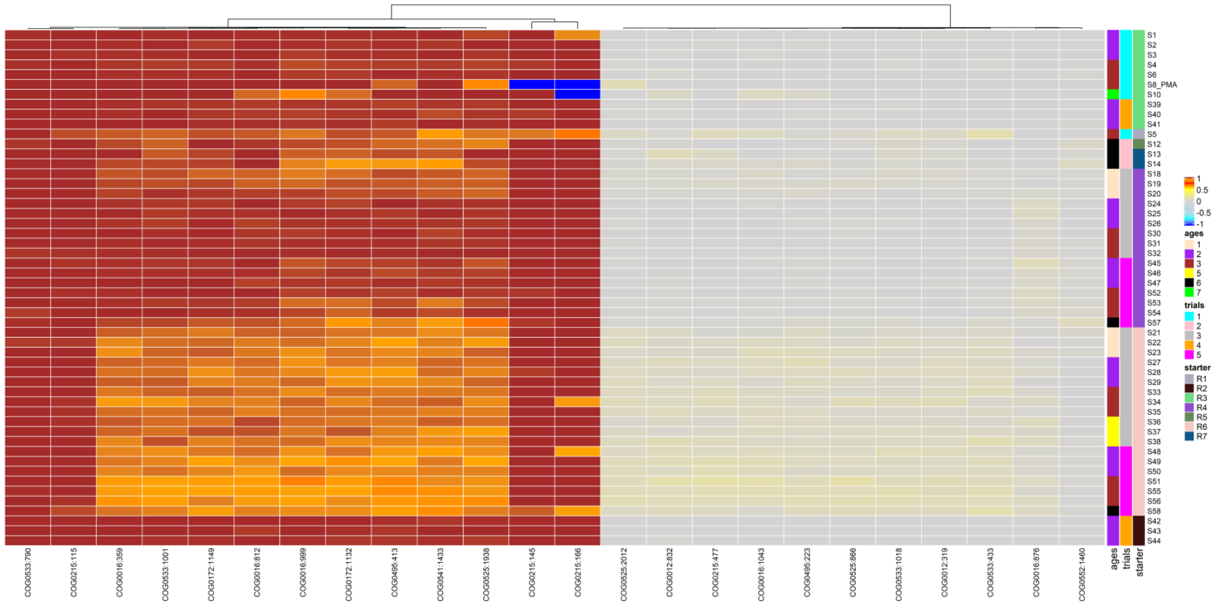

Figure S6. Heatmap of the frequencies of *Lactococcus cremoris* SNVs obtained from non-assembled reads ordered by starter, trial number and age of the cheese.

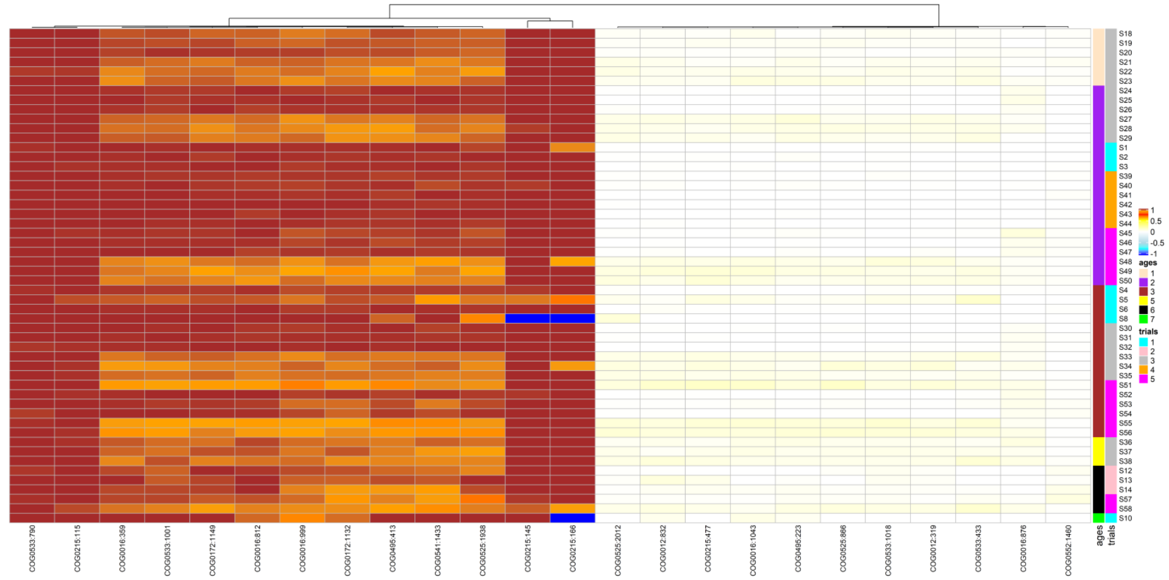

Figure S7. Heatmap of the frequencies of *Lactococcus cremoris* SNVs obtained from non-assembled reads ordered first by age of the cheese then by trial number.

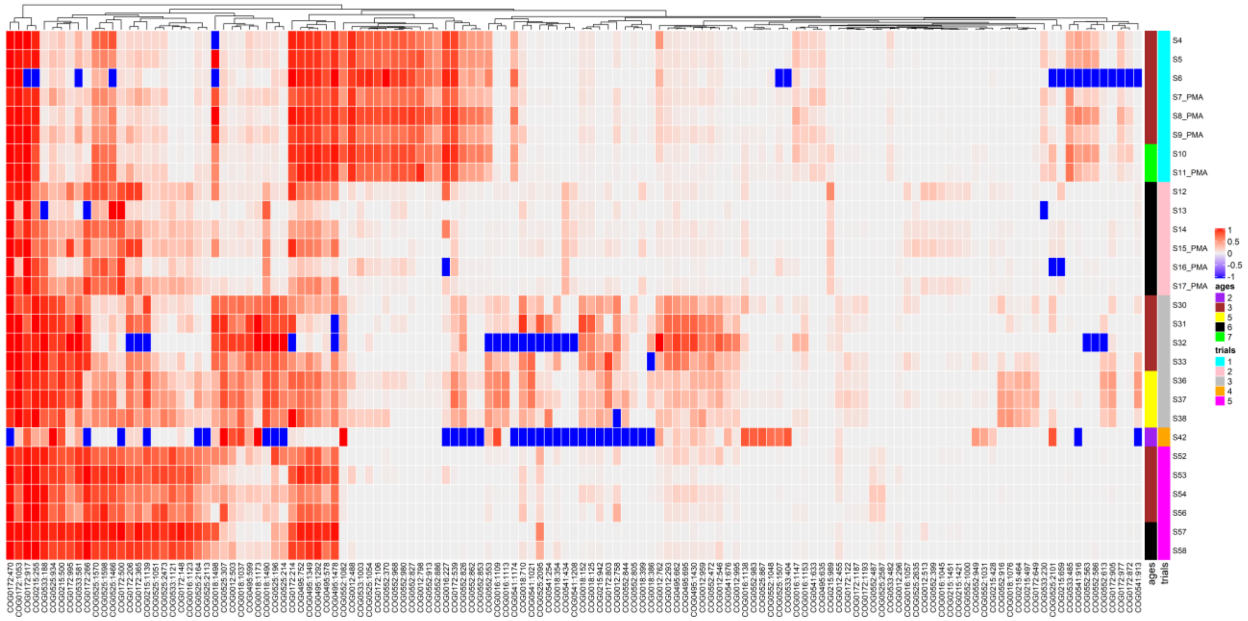

Figure S8. Heatmap of the frequency of SNVs of *Lacticaseibacillus paracasei/casei* (73 from *L. paracasei* and 62 from *L. casei*) identified from non-assembled reads ordered first by trial number and then age of cheese.

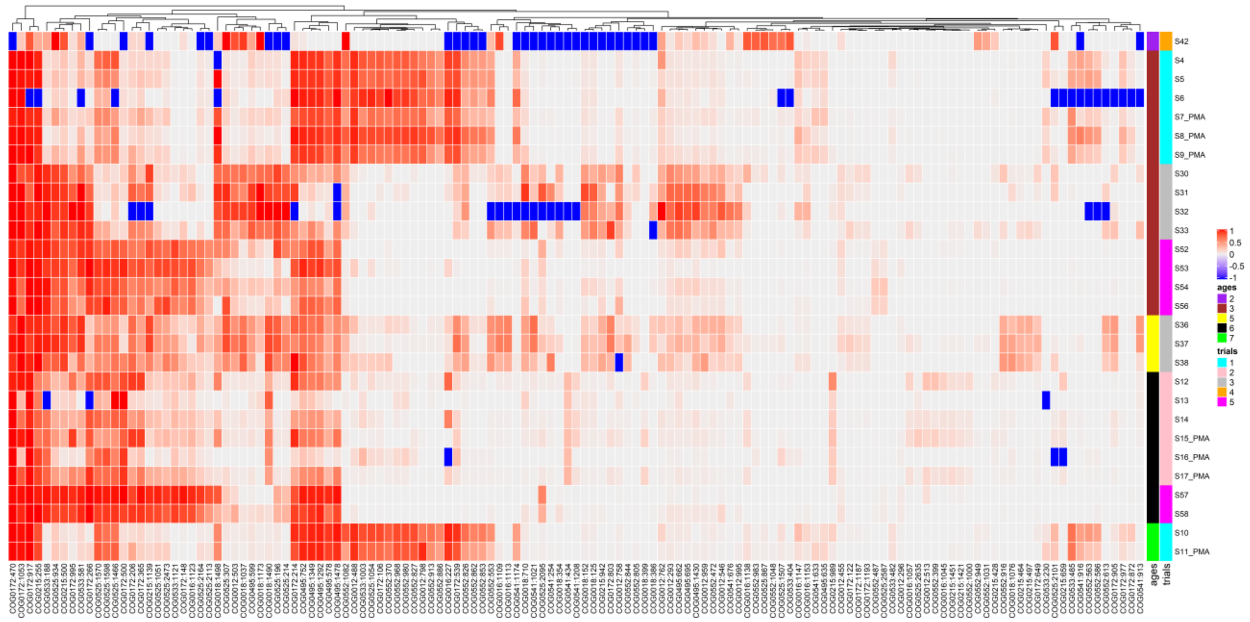

Figure S9. Heatmap of the frequency of SNVs of *Lacticaseibacillus paracasei/casei* (73 from *L. paracasei* and 62 from *L. casei*) identified from non-assembled reads ordered first by age and then by trial number.

Table S7. Breakdown of SNVs from COG0012 (predicted GTPase) that represent *L. paracasei* or *L. casei*

SNV	*L. paracasei*	*L. casei*
COG0012:296	✓	
COG0012:455	✓	
COG0012:488		✓
COG0012:503	✓	
COG0012:513	✓	
COG0012:546	✓	
COG0012:758	✓	
COG0012:959	✓	
COG0012:995	✓	
COG0012:762		✓
COG0012:798		✓

Table S8. Breakdown of SNVs from COG0016 (Phenylalanyl-tRNA synthetase alpha subunit) that represent *L. paracasei* or *L. casei*

SNV	*L. paracasei*	*L. casei*
COG0016:1045	✓	
COG0016:1057	✓	
COG0016:1109	✓	
COG0016:1113	✓	
COG0016:1123		✓
COG0016:1138		✓
COG0016:1147	✓	
COG0016:1153	✓	
COG0016:227		✓

Table S9. Breakdown of SNVs from COG0018 (Arginyl-tRNA synthetase) that represent *L. paracasei* or *L. casei*

SNV	*L. paracasei*	*L. casei*
COG0018:1498	✓	
COG0018:125		✓
COG0018:152		✓
COG0018:354		✓
COG0018:386		✓
COG0018:399		✓
COG0018:710		✓
COG0018:1037		✓
COG0018:1076		✓
COG0018:1173		✓
COG0018:1490		✓

Table S10. Breakdown of SNVs from COG00172 (Seryl-tRNA synthetase) that represent *L. paracasei* or *L. casei*

SNV	*L. paracasei*	*L. casei*
COG0172:106	✓	
COG0172:122	✓	
COG0172:148	✓	
COG0172:206		✓
COG0172:214	✓	
COG0172:365	✓	
COG0172:649		✓
COG0172:995		✓
COG0172:1053	✓	
COG0172:1187	✓	
COG0172:1193		✓
COG0172:206		✓
COG0172:266		✓
COG0172:470		✓
COG0172:500		✓
COG0172:539		✓
COG0172:803		✓
COG0172:872		✓
COG0172:905		✓
COG0172:917		✓
COG0172:977		✓

Table S11. Breakdown of SNVs from COG00215 (cysteinyl-tRNA synthetase) that represent *L. paracasei* or *L. casei*

SNV	*L. paracasei*	*L. casei*
COG0215:428	✓	
COG0215:464	✓	
COG0215:497	✓	
COG0215:500	✓	
COG0215:942	✓	
COG0215:1139	✓	
COG0215:1421	✓	
COG0215:1451		✓
COG0215:255		✓
COG0215:659		✓
COG0215:942		✓
COG0215:989		✓

Table S12. Breakdown of SNVs from COG00495 (leucyl-tRNA synthetase) that represent *L. paracasei* or *L. casei*

SNV	*L. paracasei*	*L. casei*
COG0495:578	✓	
COG0495:599	✓	
COG0495:635	✓	
COG0495:662		✓
COG0495:695	✓	
COG0495:752	✓	
COG0495:1292	✓	
COG0495:1349	✓	
COG0495:1430	✓	
COG0495:1478	✓	

Table S13. Breakdown of SNVs from COG0525 (valyl-tRNA synthetase) that represent *L. paracasei* or *L. casei*

SNV	*L. paracasei*	*L. casei*
COG0525:196	✓	
COG0525:214	✓	
COG0525:307	✓	
COG0525:867	✓	
COG0525:934	✓	
COG0525:1051	✓	
COG0525:1054		✓
COG0525:2095	✓	
COG0525:2113	✓	
COG0525:2164	✓	
COG0525:2473	✓	
COG0525:2587	✓	
COG0525:2635	✓	
COG0525:1466		✓
COG0525:1507		✓
COG0525:1570		✓
COG0525:1598		✓
COG0525:2101		✓
COG0525:2113		✓

Table S14. Breakdown of SNVs from COG0533 (metal dependent protease) that represent *L. paracasei* or *L. casei*

SNV	*L. paracasei*	*L. casei*
COG0533:404	✓	
COG0533:482	✓	
COG0533:485	✓	
COG0533:581	✓	
COG0533:188		✓
COG0533:230		✓
COG0533:1003		✓
COG0533:1121		✓

Table S15. Breakdown of SNVs from COG0541 (signal recognition particle GTPase Ffh) that represent *L. paracasei* or *L. casei*

SNV	*L. paracasei*	*L. casei*
COG0541:254	✓	
COG0541:434	✓	
COG0541:633		✓
COG0541:676	✓	
COG0541:913	✓	
COG0541:916	✓	
COG0541:1021		✓
COG0541:1174	✓	
COG0541:1208	✓	

Table S16. Breakdown of SNVs from COG0552 (signal recognition particle GTPase FtsY) that represent *L. paracasei* or *L. casei*

SNV	*L. paracasei*	*L. casei*
COG0552:370	✓	
COG0552:399	✓	
COG0552:472	✓	
COG0552:487		✓
COG0552:553	✓	
COG0552:563	✓	
COG0552:586	✓	
COG0552:613	✓	
COG0552:805		✓
COG0552:826	✓	
COG0552:844	✓	
COG0552:853	✓	
COG0552:862	✓	
COG0552:886	✓	
COG0552:913	✓	
COG0552:949	✓	
COG0552:1031	✓	
COG0552:1048		✓
COG0552:1082		✓
COG0552:827		✓
COG0552:916		✓
COG0552:968		✓
COG0552:980		✓
COG0552:983		✓
COG0552:1000		✓

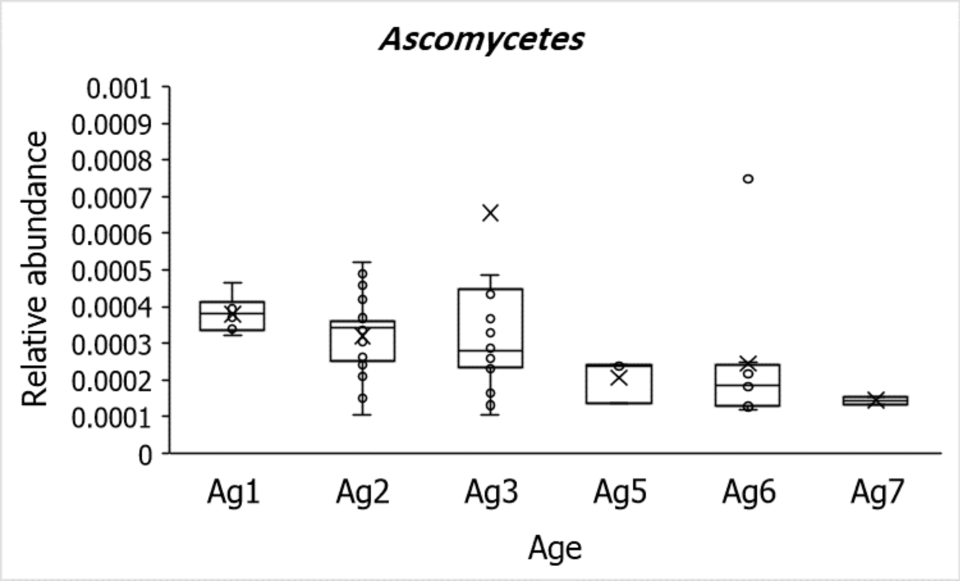

(a)

Figure S10. *Ascomycota* taxa profiling using the non-assembly-based MG-RAST approach.
